# The impact of child type 1 diabetes on parental incomes in a welfare state context: quasi-experimental evidence from Swedish national registers

**DOI:** 10.1007/s00125-025-06492-6

**Published:** 2025-08-08

**Authors:** Beatrice Kennedy, Mona-Lisa Wernroth, Sophie Langenskiöld, Carl Bonander, Liisa Byberg, Erik Grönqvist, Tove Fall

**Affiliations:** 1 Molecular Epidemiology, Department of Medical Sciences, Uppsala University, Uppsala, Sweden; 2 Uppsala Clinical Research Center, Uppsala University, Uppsala, Sweden; 3 Health Economics, Department of Medical Sciences, Uppsala University, Uppsala, Sweden; 4 Centre for Health Economic Research (HEFUU), Uppsala University, Uppsala, Sweden; 5 School of Public Health and Community Medicine, University of Gothenburg, Gothenburg, Sweden; 6 Centre for Societal Risk Research, Karlstad University, Karlstad, Sweden; 7 Medical Epidemiology, Department of Surgical Sciences, Uppsala University, Uppsala, Sweden

**Keywords:** Children, Cohort, Parents, Quasi-experimental methods, Register-based research, Socioeconomic circumstances, Sweden, Type 1 diabetes

## Abstract

**Aims/hypothesis:**

The aim of this study was to quantify the impact of childhood-onset type 1 diabetes on parental incomes in a Nordic welfare state.

**Methods:**

In this register-based quasi-experimental study, we included the parents of 13,358 children diagnosed with type 1 diabetes in Sweden from 1993 to 2014 together with 506,516 population-based matched control parents. A difference-in-differences approach was used to compare income trajectories between exposed parents and control parents. Work-related and pension-qualifying incomes (including parental benefits) were assessed during the first 7 years after diagnosis. The long-term incomes of parents of children diagnosed with type 1 diabetes in 1993–2004 were also investigated.

**Results:**

A sharp decline in work-related income was observed in both mothers and fathers of children diagnosed with type 1 diabetes. In the year after diagnosis, the mean yearly income difference (expressed in €100) was –15.4 for mothers (95% CI –17.2, –13.6) and –6.0 for fathers (95% CI –8.9, –3.2), representing a relative decrease of 6.6% and 1.6%, respectively. The effects on income were similar across sociodemographic groups and calendar periods. The pension-qualifying income of mothers increased in the first year after diagnosis by 28.7 (95% CI 27.1, 30.3), attributable to the parental care allowance, but gradually decreased during long-term follow-up (–10.9, 95% CI –16.6, –5.1, after 17 years).

**Conclusions/interpretation:**

This study highlights the enduring financial consequences for parents caring for a child with type 1 diabetes in Sweden. While parental benefits in Sweden mitigated the short-term loss of maternal income, the current welfare system does not adequately address long-term consequences.

**Graphical Abstract:**

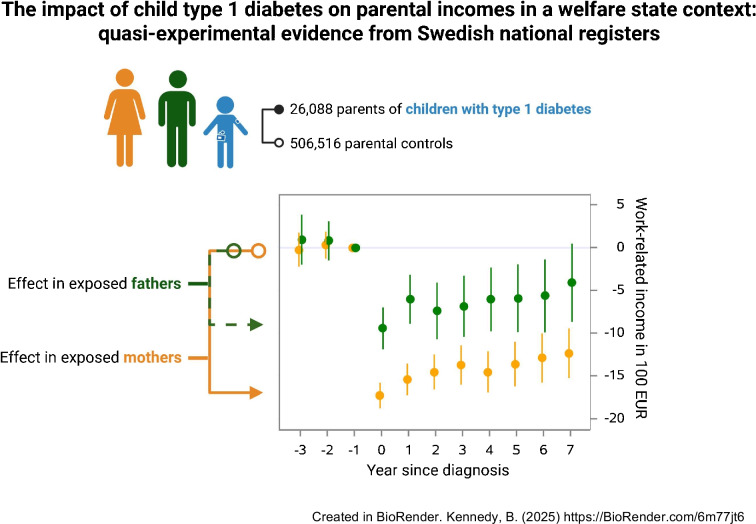

**Supplementary Information:**

The online version contains peer-reviewed but unedited supplementary material available at 10.1007/s00125-025-06492-6.


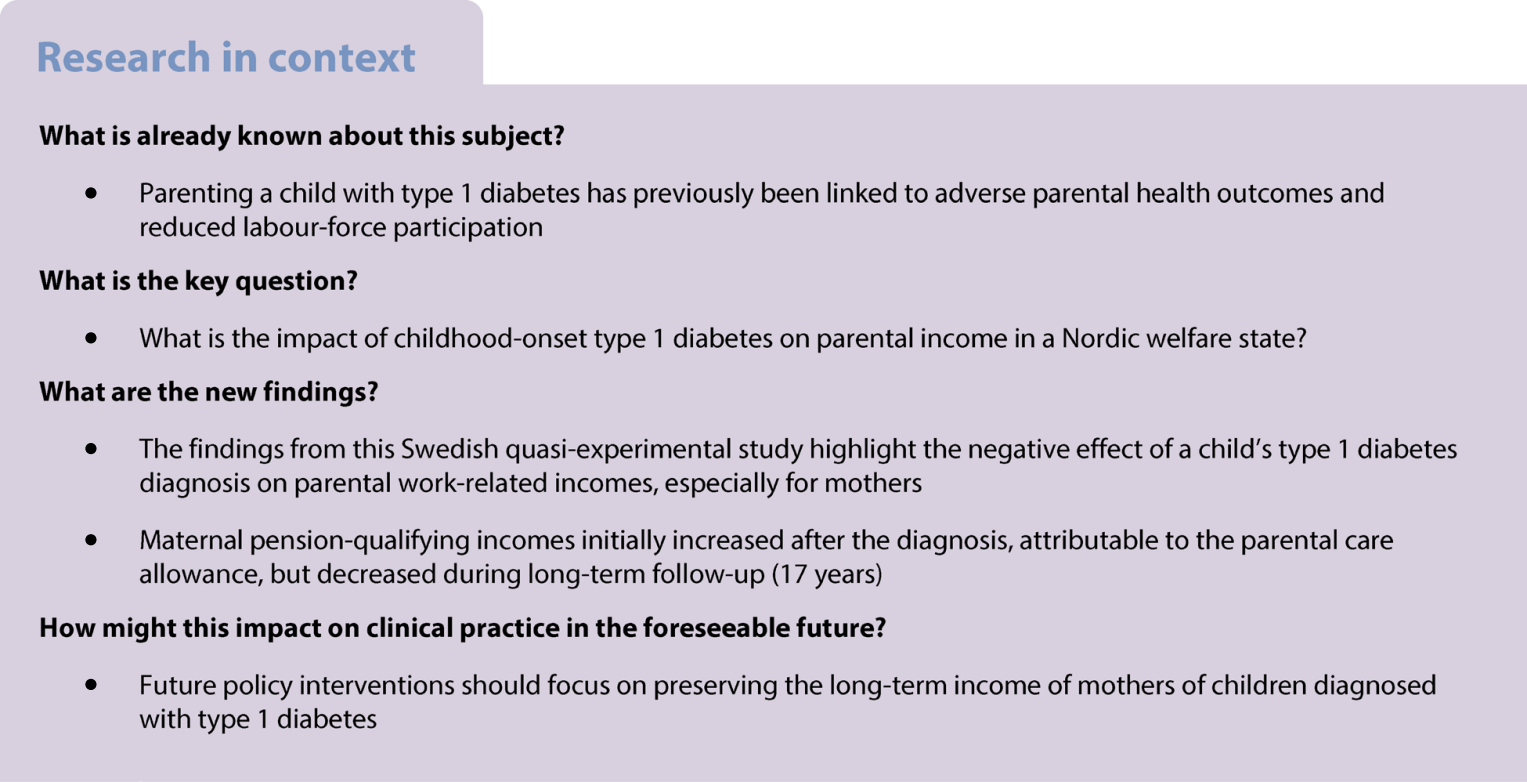


## Introduction

Parents of children with type 1 diabetes shoulder the main responsibility for childcare and disease management, including mainstay insulin administration and continuous monitoring of glucose levels [1]. Parents must also manage acute hypoglycaemic and hyperglycaemic events, uphold healthcare contacts, ensure adequate diabetes care in preschool and school settings and encourage self-efficacy in older children. Taken together, caring for a child with type 1 diabetes may impair parental labour-force participation, thereby reducing parental incomes and adding a financial strain to the burden of the disease. Elucidating such financial consequences may help strengthen future parental support strategies.

A questionnaire-based German study on the impact of parenting a child with type 1 diabetes reported that nearly half of the families suffered moderate to severe financial losses during the first year after diagnosis, primarily reflecting reduced working hours among mothers [2]. Similarly, a Danish quasi-experimental study found that in the years following a type 1 diabetes diagnosis, mothers tended to switch from full-time to part-time employment and reduced their total incomes, with no corresponding patterns identified in fathers [3]. A Swedish study of parental outcomes during early childhood of children with chronic conditions, mainly neurodevelopmental disorders, found that mothers had lower incomes and a subsequent greater increase in risk of early retirement due to sickness or disability than fathers [4].

The incidence rate of childhood-onset type 1 diabetes in Sweden has increased considerably over the past few decades, reaching 41.4 cases per 100,000 person-years in 2021 (the second highest rate across the globe) [5]. Sweden is further recognised as a strong welfare state with universal healthcare, a social security system that includes parental care benefits and one of the world’s highest maternal employment rates [6]. However, the short- and long-term effects of childhood-onset type 1 diabetes on maternal and paternal incomes in this setting, and the extent to which social security benefits compensate for any loss of work-related income, have not been elucidated. In this study, we assessed work-related and pension-qualifying income trajectories of mothers and fathers of children with type 1 diabetes in comparison with population-based matched controls. We leveraged prospectively collected data from the national population and health registers in Sweden, using a difference-in-differences (DiD) approach and applying recent methodological advances [7, 8] to control for time-invariant unobserved confounding and to estimate the effects on income relative to the year before the child’s type 1 diabetes diagnosis.

## Methods

### Study population, exposure and population-based controls

Detailed information on the study population has been published [9]. The source population comprised parents in Sweden with biological children born between 1987 and 2020. The analytical dataset was constructed by linkages across national population registers held by Statistics Sweden, national health registers held by the National Board of Health and Welfare, and the National Child Diabetes Register (Swediabkids). This study was approved by the Swedish Ethical Review Authority (DNR 2020-02206 with addenda 2020-04497 and 2021-01956).

Exposure was defined as having a biological child diagnosed with type 1 diabetes. All children born between 1987 and 2014 who were diagnosed with type 1 diabetes (at <18 years of age) from 1993 to 2014 in Sweden were included. Child type 1 diabetes was defined as an inpatient diagnosis in the National Patient Register (NPR; ICD-9 code 250 or ICD-10 code E10), a prescription of insulin in the Swedish Prescribed Drug Register (SPDR; Anatomical Therapeutic Chemical code A10A), or a diagnosis of type 1 diabetes in Swediabkids (additional details available in the Electronic Supplementary Material [ESM] Methods: Type 1 diabetes across registers).

The date of diagnosis (the index date) was defined as the date of the first diagnosis in any of these registers. The biological parents of the children were identified through the Multi-Generation Register.

Twenty control parents for each exposed parent, selected from the Swedish Total Population Register, were matched on the registered sex and year of birth of the parents and the year of birth of the child. This approach allowed the study population to be considered representative of the Swedish parent population during the study period. After exclusions [9], the study population comprised 26,088 exposed parents and 506,516 control parents.

### Outcomes

Data on the annual gross incomes of each parent in Swedish krona (SEK; €100=1160 SEK [10]) were obtained from annual excerpts from the Longitudinal Integrated Database for Health Insurance and Labor Market Studies (LISA), available from 1990 onwards [11]. LISA retrieves information from the Swedish Tax Agency, which collects mandatory income reports from all companies and employers in Sweden, and from the Swedish Social Insurance Agency. LISA includes complete income information on all adult residents of Sweden. We used data from 1990 to 2021. All incomes were adjusted for annual inflation using the Consumer Price Index, the standard measure of inflation calculation in Sweden [12], and were converted to 2024 price levels.

#### Work-related income

The first main outcome was work-related income. This variable summarises taxed income from employment and self-employment and reflects an individual’s monthly or hourly salary multiplied by the extent of the employment (full-time or part-time).

#### Pension-qualifying income

The second main outcome was pension-qualifying income. In Sweden, work-related incomes, parental benefits, sick-leave benefits, disability allowances and unemployment benefits constitute pension-qualifying income on which future old-age pensions are based [13]. Incomes from old-age pension, social welfare or capital gains from stocks, bonds or property sales are non-pension-qualifying.

Parental benefits, available for both mothers and fathers in Sweden, include parental leave, temporary care for a sick child, parental care allowance and municipality care allowance. The level of compensation from all parental benefits (except the municipality care allowance) depends on parental work-related income. Parental leave currently comprises 480 days, which can be used by either parent from around the birth of a child until age 12 years (8 years for children born before 2014). Temporary care for a sick child is a compensatory parental benefit for parents who temporarily miss work or refrain from seeking unemployment benefits to care for a sick child. This benefit mainly applies to parents of children from the age of 8 months up to and including the day the child turns 12 years but may also apply to parents of children aged ≥12 but <16 years who have a severe medical condition or a chronic disease, including type 1 diabetes. The maximum number of days for temporary care for a sick child is 120 per child per calendar year, which the parents can split. Parental care allowance is a monthly benefit applicable to parents of a child with certain specific chronic conditions or special needs that are expected to require a high and continuous level of parental care or supervision, including type 1 diabetes. Parents of younger children generally receive higher parental care allowance compensation than parents of older children. Municipality care allowance, available from 2008 to 2016, was a low-level compensation available in fewer than half of the Swedish municipalities to parents of children aged 1–2 years who did not attend preschool, regardless of child health conditions.

Sick-leave benefits include sick pay, compensation for work-related illness and rehabilitation compensation. Sick-leave pay and other sickness benefits for leave episodes >14 days are administered by the Swedish Social Insurance Agency. Disability allowance compensates for part-time or full-time early retirement due to sickness or disability. Unemployment benefits are available for individuals who have previously worked or been self-employed.

### Parental baseline characteristics

Parental sociodemographic variables, extracted from the Total Population Register and LISA, included country of birth (categorised as Sweden, other European countries and non-European countries), highest education level (compulsory, secondary or university) and marital status (single, married or cohabitating with joint children). Baseline characteristics were assessed on 31 December of the calendar year preceding the index year.

Parental type 1 diabetes was defined as a diagnosis of type 1 diabetes (main or secondary inpatient diagnosis or main outpatient diagnosis) in the NPR and/or a diagnosis of type 1 diabetes in Swediabkids at <18 years of age, at index date.

### Statistical analysis

All analyses were done with R software (version 4.2.3, March 2023) [14]. To investigate the impact of having a child diagnosed with type 1 diabetes on parental incomes, we used a DiD approach. This quasi-experimental event study method can be used to estimate the impact of interventions (natural or experimental) [15, 16]. Here, the natural intervention was the diagnosis of a child with type 1 diabetes. The year of diagnosis constituted the index year (t = 0) for the parents of children with type 1 diabetes and the matched control parents. Fixed-effects linear regression, including an interaction with index year, was applied to avoid potential bias from variation in exposure timing and effect heterogeneity [8]. Separate regression models were fitted for mothers and fathers, and effect estimates for each follow-up year are reported. Follow-up ended at parental death, and the incomes of emigrated parents were set to missing. In addition, the average post-exposure effect on maternal and paternal incomes was compared by fitting a model that included a multiplicative interaction between parents’ sex and exposure. SEs were clustered at the individual level in all models to account for within-individual dependence over time, as detailed in the ESM Methods. The output was the mean effect of the intervention per €100 presented with 95% CIs.

A critical assumption of the DiD method is that the groups would have had parallel outcome trends without the intervention. In practice, this is often assessed by inspecting whether the trends in the period before the intervention are parallel. Our analyses therefore included parental income data from 3 years before and 7 years after the index year. Since the DiD builds on investigating the difference between groups after the event compared with the difference between the groups before the event, the groups are not assumed to be similar in time-invariant covariates or in factors that change equivalently in both groups over time. Thus, no additional adjustments were required, for example, for parental type 1 diabetes status or country of birth. If a parent had more than one child diagnosed with type 1 diabetes, the year of diagnosis of the first child was used as the index year. We also conducted two sensitivity analyses. In the first, we excluded all parents diagnosed with type 1 diabetes before the index date and in the second, we censored parents if they had a second child diagnosed with type 1 diabetes.

In six subgroup analyses, we determined whether parental work-related income patterns differed by the sex or age of the child at the index year, the index year calendar period (1993–2000, 2001–2007, 2008–2014) or the country of birth, education level or marital status of the parent. Estimates are presented for each year of follow-up but a simplified model that only compares the post-exposure mean effect was used to test for differences between subgroups. We added one multiplicative interaction at a time to the model, applied the Wald test and considered a *p* value of <0.05 statistically significant. Participants with missing values for sociodemographic variables were excluded from the respective subgroup analyses.

We conducted two post hoc analyses on work-related income. In the first, we examined whether the impact on maternal work-related income varied depending on the maternal contribution to the household’s total disposable income. We calculated the maternal share of the household’s total income using the LISA variables disposable individual income and disposable familial income, the latter reflecting the combined disposable income of all adults in the household. Disposable income is defined as total income after taxes. It includes pension-qualifying incomes together with incomes from other sources such as old-age pensions, social welfare benefits and capital gains from stocks, bonds or property sales. This analysis included mothers who were married or cohabiting with joint children in the year before the index year. Of the 216,278 married or cohabiting mothers, 550 were excluded due to negative disposable household income, typically resulting from capital losses. We categorised mothers by their contribution to the family disposable income the year preceding the index year (<50% and ≥50%; 77% and 23% of mothers, respectively). In the second post hoc analysis, we assessed whether the estimates of work-related income in mothers and fathers differed by the number of children registered in the household the year preceding the index year (≤1 or ≥2).

To investigate the potential long-term effects on work-related income, we analysed a subcohort of parents with an index year from 1993–2004, which enabled a follow-up of up to 17 years.

To assess the impact on the pension-qualifying income, we applied the same regression models used for work-related income to the full study population and the subcohort with an index year in 1993–2004. Since we detected a notable increase in pension-qualifying income in mothers, we performed post hoc analyses of pension-qualifying income excluding the parental care allowance.

Lastly, to assess the pre-parallel trend assumption for a longer 7 year pre-period, we performed a sensitivity analysis restricted to parents with an index date in 1997–2014.

## Results

Baseline characteristics for parents of children with type 1 diabetes and the population-based matched control parents are presented in ESM Table 1. The median age at the index date was 38 years in mothers and 41 years in fathers. Type 1 diabetes was more than five times more prevalent in exposed parents than in control parents (3.5% vs 0.6% of mothers and 5.2% vs 0.9% of fathers). Further, parents of children with type 1 diabetes were more often born in Sweden. Residential characteristics were similar across the exposure groups.

The median age of type 1 diabetes diagnosis in children was 9.0 years (IQR 5.4–12.4; ESM Table 2). Children diagnosed with type 1 diabetes (*n*=13,358) were more often boys and more often born in Sweden than the children of the control parents.

In the year before the index year, the absolute work-related incomes of mothers were markedly lower than those of fathers, regardless of exposure status, with mothers earning less than three-fifths of the fathers’ income. The yearly mean work-related incomes (in €100) of later-exposed parents were slightly higher than those of the control parents (202 vs 196 for mothers and 349 vs 341 for fathers). Similarly, the absolute pension-qualifying incomes of mothers were lower than those of fathers, regardless of exposure status, in the year before the index year. The yearly mean pension-qualifying incomes of later-exposed parents were again somewhat higher than those of the controls (244 vs 235 for mothers and 372 vs 364 for fathers) in the year before the index year. We noted parallel pre-trends before index for both types of incomes.

### Work-related incomes

**Fig. 1:**
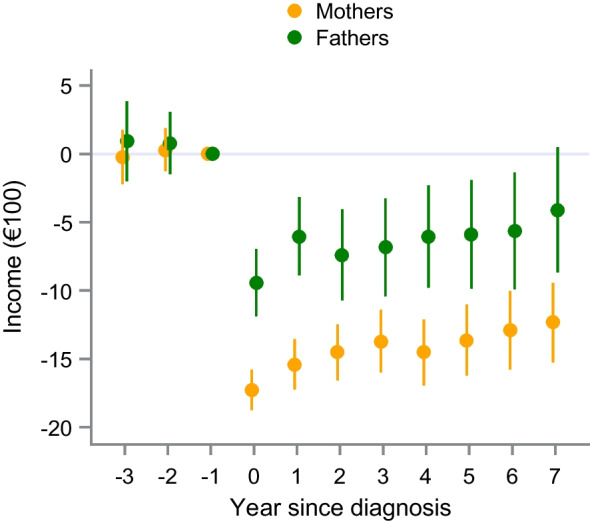
Yearly effect estimates from the DiD analysis (mean differences and 95% CIs) of work-related incomes in mothers and fathers of children with type 1 diabetes. Index years 1993–2014. Incomes are reported in €100

In the main DiD analysis, we observed a drop in work-related incomes of both mothers and fathers of a child diagnosed with type 1 diabetes, as compared with the year before the index year (Fig. 1 and ESM Table 3). The reduction was larger (*p*_int_<0.001, ESM Table 4a) and more persistent in exposed mothers than in exposed fathers. In the first year after the index year, the mean difference was (in €100) −15.4 (95% CI −17.2, −13.6) in mothers and −6.0 (95% CI −8.9, −3.2) in fathers, representing a relative decrease of 6.6% and 1.6%, respectively. After 7 years, the respective estimates were −12.3 (95% CI −15.3, −9.4) and −4.1 (95% CI −8.7, 0.5), or relative decreases of 3.9% and 0.9%. The sensitivity analysis excluding parents with type 1 diabetes at index yielded similar estimates (ESM Table 5). Overall, 508 of the 13,182 exposed mothers and 497 of the 12,906 exposed fathers had a second child diagnosed with type 1 diabetes during the study period. The estimates of a sensitivity analysis censoring these parents at the second child’s diagnosis also closely resembled the main results (ESM Table 6).

**Fig. 2:**
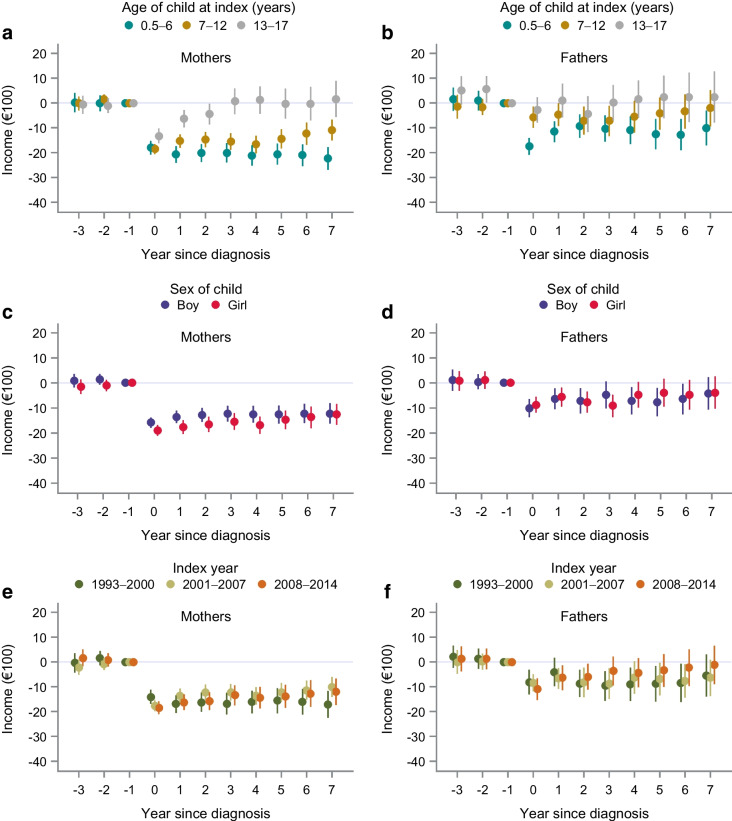
Yearly effect estimates from the DiD analysis (mean differences and 95% CIs) of work-related incomes in mothers (**a**, **c**, **e**) and fathers (**b**, **d**, **f**) of children with type 1 diabetes in subgroup analyses of age of child at diagnosis (**a**, **b**), sex of child (**c**, **d**) and index period (**e**, **f**). Index years 1993–2014. Incomes are reported in €100

**Fig. 3:**
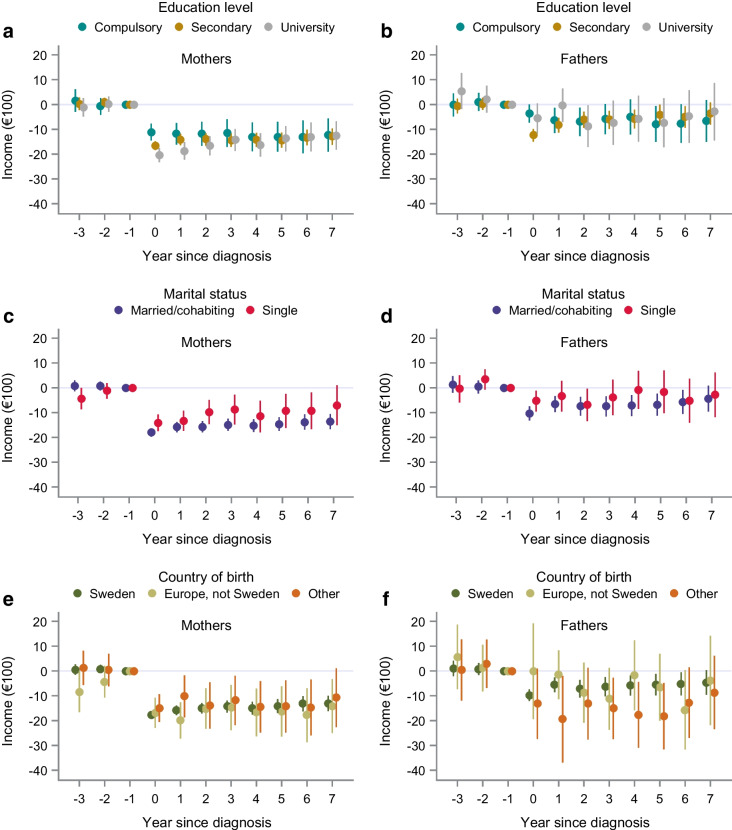
Yearly effect estimates from the DiD analysis (mean differences and 95% CIs) of work-related incomes in mothers (**a**, **c**, **e**) and fathers (**b**, **d**, **f**) of children with type 1 diabetes in subgroup analyses across parental education level (**a**, **b**), marital status (**c**, **d**) and country of birth (**e**, **f**). Index years 1993–2014. Incomes are reported in €100

In subgroup analyses, the estimated average effects on work-related income among both mothers and fathers differed by age of child at index (*p*_int _< 0.001 and *p*_int _= 0.005, respectively; ESM Table 4b). For mothers, a diagnosis of a child with type 1 diabetes at age 13–17 years had little effect on work-related income except for in the first few years after diagnosis (Fig. 2a, b and ESM Table 7). However, we noted lasting decreases in income of mothers of children diagnosed at age 7–12 years, with an even more prominent impact in mothers of children diagnosed at age ≤6 years. A similar but less pronounced pattern was found in fathers across the age groups. When examining the baseline characteristics of parents by child age groups (ESM Table 8), we observed that the age of the child at index was positively associated with parental age. We could detect no differences in the effect on work-related income due to the sex of the child, index year period, education level or country of birth (Figs 2, 3 and ESM Tables 7, 9). Overall heterogeneity was observed in maternal marital status (*p*=0.01) but the estimates had overlapping CIs.

In post hoc analyses, we observed somewhat larger effect estimates in mothers who contributed with ≥50% of the family disposable income before index, compared with mothers who contributed with <50% (ESM Table 10). However, the relative income drops were similar (6.3% and 7.0%, respectively, in the first year after index). We further noted slightly larger effect estimates in mothers with two or more children registered in the household the year before the index year, than in mothers with fewer children at home (*p*_int _= 0.04; ESM Table 11, ESM Fig. 1). No such pattern was detected in fathers (*p*_int _= 0.15).

**Fig. 4:**
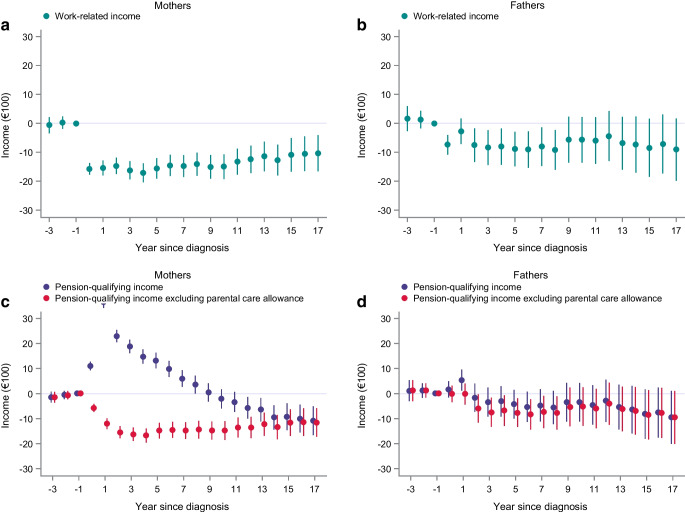
(**a**, **b**) Yearly effect estimates from the DiD analysis (mean differences and 95% CIs) of work-related incomes in mothers (**a**) and fathers (**b**) of children with type 1 diabetes. (**c**, **d**) Yearly effect estimates from the DiD analysis (mean differences and 95% CIs) of pension-qualifying incomes in mothers (**c**) and fathers (**d**) of children with type 1 diabetes, with and without parental care allowance. Index years 1993–2004. Incomes are reported in €100

To explore the effects on work-related income beyond 7 years, we analysed parents with an index date in 1993–2004 (baseline characteristics of parents and children are shown in ESM Tables 12 and 13, respectively) with a longer follow-up period. Because the sample size was smaller, this analysis rendered less precise estimates. However, the larger impact on maternal work-related income persisted across the longer follow-up, with a mean difference (in €100) after 17 years of −10.3 (95% CI −16.6, −4.1; relative decrease 3.1%; Fig. 4a and ESM Table 14). The overall smaller negative estimates in fathers also lingered over time but with large CIs, with a mean difference after 17 years of −9.1 (95% CI −19.9, 1.7; relative decrease 2.2%; Fig. 4b and ESM Table 14).

### Pension-qualifying incomes

We investigated pension-qualifying incomes to elucidate any compensatory effects of social security and parental care benefits. In contrast to our findings on work-related incomes, our DiD analysis showed elevated levels of pension-qualifying incomes in exposed mothers across the 7 years of follow-up, with the highest mean differences (in €100) noted the year after index (28.7, 95% CI 27.1, 30.3; ESM Fig. 2, ESM Table 15). No such pattern was noted for fathers, and the post-exposure average effect was larger for mothers than for fathers (*p*_int _< 0.001, ESM Table 4a). Analysis of long-term effects (index years 1993–2004) revealed an analogous maternal pattern until 8 years after the index year (Fig. 4c and ESM Table 16), when the maternal pension-qualifying incomes started to decrease, and at the end of the 17-year follow-up they were lower than in the unexposed mothers (−10.9, 95% CI −16.6, −5.1). The lower paternal pension-qualifying estimates also persisted over time but with larger CIs (after 17 years: −9.5, 95% CI −20.1, 1.1; Fig. 4d and ESM Table 16).

In a post hoc analysis, in which we excluded the parental care allowance from the pension-qualifying incomes, we observed overall negative estimates for the pension-qualifying incomes in mothers, similar to the lower estimates of work-related income (Fig. 4c, d and ESM Fig. 2, ESM Tables 17, 18).

Lastly, the sensitivity analysis restricted to parents with an index date in 1997–2014 indicated parallel pre-trends up to 7 years before the index year for all types of incomes (work-related incomes, pension-qualifying incomes and pension-qualifying incomes excluding the parental care allowance [ESM Fig. 3]).

## Discussion

In this population-based study, parenting a child diagnosed with type 1 diabetes was linked to a sharp decline in work-related income. The decrease was more persistent in mothers and represented a larger proportion of their earnings than for fathers. In contrast, maternal pension-qualifying incomes increased after a child’s diabetes diagnosis, attributable to the parental care allowance. However, during the second half of the 17 year follow-up period, maternal pension-qualifying incomes gradually decreased and had not recovered by the end of the study period.

### Strengths and limitations

The strengths of this study include the population-based design, the prospectively collected high-quality health data and complete income register data, and the ability of the DiD design to account for both observable and unobservable time-invariant confounders such as parental country of birth or genetic predisposition to develop autoimmune conditions. Our study had several limitations. First, we could not fully ascertain the parents’ employment type, weekly workload or other occupational circumstances. We detected no differences across education levels but our register data did not enable us to investigate, for example, whether work flexibility could modify the impact on work-related incomes. Second, during the study period, child healthcare, insulin and diabetes devices were heavily subsidised or provided free of charge in Sweden. The baseline maternal employment rates in Sweden are high and mothers and fathers are equally eligible for parental benefits. Thus, our findings may be relevant only to other welfare states with similar social security system characteristics. Third, although DiD analysis controls for time-invariant confounders, our estimates may be biased by non-parallel counterfactual trends, which cannot be tested after the intervention. However, our pre-trend assessments showed limited cause for concern, even in the sensitivity analysis with an extended pre-time period of 7 years.

### Comparisons with previous studies

While our findings largely aligned with previous studies on the impact of incomes on parents of children with type 1 diabetes, some differences should be emphasised. In a German study of 1144 families that showed reduced maternal labour-force participation after a child’s type 1 diabetes diagnosis, 91% of fathers but only 23% of mothers worked full-time in the year before diagnosis [2], reflecting a larger underlying gender inequality gap in Germany than in Sweden [17]. A Danish study of parents of 5838 children diagnosed with type 1 diabetes in 1990–2007, which also used a DiD design, reported a 4–5% maternal decrease in work-related income during the end of the 10 year follow-up, similar to our relative results in mothers [3]. However, that study detected no impact on paternal incomes except for 2–4 years after diagnosis, although this may partly be due to lower statistical power. In our study, the work-related income of exposed fathers was reduced over a longer period.

Parenting a child diagnosed at preschool age had a greater impact on parental work-related incomes than having a child diagnosed later in childhood. Overall, mothers of younger children in Sweden account for the majority of parental leave and temporary care of a sick child, and they are more likely to work part-time [18, 19]. Our findings suggest that this gender imbalance is magnified in parents of children with type 1 diabetes. In Sweden, all children aged >12 months are entitled to publicly funded preschool [20, 21]. Preschool is heavily subsidised and has, since 2001, had capped fees to promote enrolment. There are no additional costs for children requiring special needs support or medical supervision. The only targeted compensation to parents of children not attending preschool during our study period was the municipality care allowance. This was available in fewer than half of the Swedish municipalities between 2008 and 2016 to parents of children aged 1–2 years who did not attend preschool, and uptake was low [22]. It is thus unlikely that preschool fees or other targeted benefits to stay-at-home parents constituted major incentives for mothers of children with type 1 diabetes to reduce their working hours.

We also examined pension-qualifying incomes, which reflect the incomes that determine future old-age pensions and include the societal targeted support for parents of children with chronic illnesses. We found that maternal pension-qualifying incomes rose in the years after the index year, attributable to mothers receiving the parental care allowance. The parental care allowance is intended to compensate for the loss of work-related income and to contribute toward disease-specific costs. Our findings suggest that the parental care allowance more than compensated for the short-term loss of maternal work-related income, although we cannot discern the proportion of the allowance used to cover costs associated with type 1 diabetes. Nevertheless, maternal work-related and pension-qualifying incomes were markedly reduced in the longer follow-up, suggesting a lasting effect on maternal income trajectories even after the child reached adulthood. A similar but less pronounced and precise pattern of pension-qualifying incomes emerged for fathers in the main analysis and the longer follow-up. Our results suggest that parenting a child diagnosed with a chronic disease mid-career slows the accumulation of human capital, possibly more so if the parent and the child are younger when the child is diagnosed.

Eligibility for the parental care allowance evolved during our study period. Starting in 2019, both parents could apply for the allowance, whereas before that year, only one parent could apply. However, the vast majority of the recipients of the parental care allowance were mothers, both before and after 2019 [23], suggesting that the gender differences in pension-qualifying incomes we observed remain relevant. We have previously reported that mothers of children with type 1 diabetes may be at higher risk of early development of ischaemic heart disease [9], and long-term effects on income could be partly mediated or exacerbated by adverse somatic health outcomes associated with parenting stress.

Overall, our results emphasise the financial impact of caring for a child diagnosed with type 1 diabetes. However, the parenting stress and the effects on parental labour-force participation may be similar across a range of childhood-onset chronic conditions and we propose that our findings could serve as a model to elucidate the financial consequences for parents caring for a child with chronic disease in a Nordic welfare state.

In conclusion, in this large population-based study, we found that parenting a child diagnosed with type 1 diabetes yielded long-term negative effects on parental work-related incomes. Parental benefits mitigated the short-term loss of maternal income but the current welfare system did not offset the adverse long-term income consequences. These findings underscore the need for sustained societal efforts to promote gender equity in both the labour market and in the care of children, as we believe that it is the underlying gender norms that may be amplified in parents of children with type 1 diabetes. Future policy initiatives should further consider targeted support to parents of children with severe chronic disease to alleviate the lasting gender imbalances in incomes, which may promote the economic stability and the health of mothers and their children.

## Supplementary Information

Below is the link to the electronic supplementary material.

ESM (PDF 777 KB)

## Data Availability

Restrictions apply to the availability of the data, which were used under license and ethical approval and are not publicly available. However, data are available from the corresponding author upon reasonable request and with written permission of the Swedish Ethical Review Authority, subject to legal contracts regarding the General Data Protection Regulation (GDPR) and Personal Data Processing Agreements between Uppsala University and the receiving research entity.

## Abbreviations

DiD: Difference-in-differences
LISA: Longitudinal Integrated Database for Health Insurance and Labor Market Studies
NPR: National Patient Register
SEK: Swedish krona
