# Supplementary material for: The impact of child type 1 diabetes on parental incomes in a welfare state context: quasi-experimental evidence from Swedish national registers
Source: Diabetologia. 2025 Aug 8;68(10):2168–78. doi: 10.1007/s00125-025-06492-6 (PMC12423126; doi:10.1007/s00125-025-06492-6)
Supplement: Supplementary file 1 — ESM (PDF 777 KB) [file 125_2025_6492_MOESM1_ESM.pdf]

ELECTRONIC SUPPLEMENTARY MATERIAL (ESM)

Type 1 diabetes across registers

The National Patient Register contains data on the main and secondary diagnoses of all inpatients in Sweden from 1987 onward. During our study period, the national recommendation was to hospitalize all children upon diagnosis of type 1 diabetes, regardless of the initial general condition. Children diagnosed in other countries will not have an inpatient diagnosis of type 1 diabetes unless they were hospitalized in Sweden for type 1 diabetes or any other condition after immigration. The Swedish Prescribed Drug Register contains information on all dispensed prescriptions of pharmacological agents, including insulin, in Sweden from 2005 and onwards. The quality register Swediabkids was founded in 2020, and in 2007, all pediatric diabetes clinics across the country were enrolled to report data, including the date of diagnosis. The coverage of all children with type 1 diabetes in Swediabkids was estimated to be 92.7% in 2019, with regional differences in coverage across Sweden ranging from 77.5% to 98.7%. [1]

Statistical analysis

We defined the index date as the date of diagnosis of type 1 diabetes in children to exposed parents, and we set the same date as the index date in the matched parental controls. After that, a yearly panel starting 3 years before the index year and ending 7 years after was constructed, and a difference-in-differences event study method was applied using fixed effects linear regression. Separate regression models were fitted for the different types of outcomes and mothers and fathers.

The models (1), where y_{it} denotes the outcome for parent i t years from index (t=-3, -2, -1, 0, 1, 2, 3, 4, 5, 6, 7), included individual fixed effects (α_i) and two interactions ($\beta_{j,k}$ and $\gamma_{j,k}$)

$$y_{it} = \alpha_i + \sum_{j\neq-1}\sum_k \beta_{j,k}\,\mathrm{I}(\mathrm{j}=\mathrm{t})\mathrm{I}(\mathrm{k}=\mathrm{p}) + \sum_{j\neq-1}\sum_k \gamma_{j,k}\,\mathrm{I}(\mathrm{j}=\mathrm{t})\mathrm{I}(\mathrm{k}=\mathrm{p})\mathrm{I}(\mathrm{Exposed}) + \varepsilon_{it} \quad (1)$$

The Is are indicator variables. For time-to-index the reference group was -1. For exposure, the reference group was the matched controls. $\beta_{j,k}$ represents the interaction between the two factors; time-to-index(t) and index year (p). $\gamma_{j,k}$ represents the interaction between the

three factors; time-to-index (t), index year(p) and exposure. The coefficients $\gamma_{i,k}$ are estimates of the difference between exposed and controls, relative to the year before diagnosis (t=-1), for each possible combination of time-to-index and index year. Furthermore, we calculated an average for each of the three years before the index year and the seven years after (10 years in total), using the frequency distribution of index years in the exposed group as weights.

INDEX ELECTRONIC SUPPLEMENTARY TABLES AND FIGURES

ESM Table 1. Baseline characteristics of mothers and fathers of children with type 1 diabetes (exposed) and their population-based controls (unexposed). Parental type 1 diabetes was assessed at the index date. Index years 1993-2014. 5

ESM Table 2. Baseline characteristics of the children diagnosed with type 1 diabetes and the children of the population-based matched parental controls. Index years 1993-2014. 7

ESM Table 3. Mean absolute, mean differences and 95% CIs, and relative differences in work-related incomes in mothers and fathers of children with type 1 diabetes (exposed) and their population-based matched controls (unexposed). Index years 1993-2014. Yearly incomes are reported in €100. 8

ESM Table 4. P-values for the interaction of average treatment effect across 7 years in a) mothers versus fathers, and b) in subgroup analyses. 9

ESM Table 5. Sensitivity analysis excluding all parents diagnosed with type 1 diabetes at index date. Mean differences and 95% CIs of work-related incomes in parents of children with type 1 diabetes (exposed) and the population-based matched parental controls (unexposed). Index years 1993-2014. Yearly incomes are reported in €100. 10

ESM Table 6. Sensitivity analysis censoring exposed parents if a second child is diagnosed with type 1 diabetes. Mean differences and 95% CIs of work-related incomes in parents of children with type 1 diabetes (exposed) and the population-based matched parental controls (unexposed). Index years 1993-2014. Yearly incomes are reported in €100. 11

ESM Table 7. Subanalyses across child characteristics and calendar periods. Mean differences and 95% CIs of work-related incomes in mothers (a) and fathers (b) of children with type 1 diabetes (exposed) and the population-based matched parental controls (unexposed). Index years 1993-2014. Yearly incomes are reported in €100. 12

ESM Table 8. Baseline characteristics of mothers (a) and fathers (b) of children with type 1 diabetes and their population-based controls, categorized by age of child at index. Parental type 1 diabetes was assessed at the index date. Index years 1993-2014. 14

ESM Table 9. Subanalyses across parental sociodemographic characteristics. Mean differences and 95% CIs of work-related incomes in mothers (a) and fathers (b) of children with type 1 diabetes (exposed) and the population-based matched parental controls (unexposed). Index years 1993-2014. Yearly incomes are reported in €100. 18

ESM Table 10. Post hoc analysis. Mean absolute, mean differences and 95% CIs, and relative differences in work-related incomes in mothers and fathers of children with type 1 diabetes (exposed) and their population-based matched controls (unexposed), by the maternal proportion of familial disposable income the year preceding the index year. Index years 1993-2014. Yearly incomes are reported in €100. 20

ESM Table 11. Post hoc analysis. Mean differences and 95% CIs of work-related incomes in parents of children with type 1 diabetes (exposed) and the population-based matched parental controls (unexposed), by the number of children registered in the parental household the year preceding the index year. Index years 1993-2014. Yearly incomes are reported in €100. 21

ESM Table 12. Baseline characteristics of mothers and fathers of children with type 1 diabetes (exposed) and their population-based controls (unexposed). Parental type 1 diabetes was assessed at the index date. Index years 1993-2004. 22

ESM Table 13. Baseline characteristics of the children diagnosed with type 1 diabetes and the children of the population-based matched parental controls. Index years 1993-2004. 24

ESM Table 14. Mean absolute, mean differences and 95% CIs, and relative differences in work-related incomes in mothers and fathers of children with type 1 diabetes (exposed) and the population-based matched parental controls (unexposed). Index years 1993-2004. Yearly incomes are reported in €100. 25

ESM Table 15. Mean absolute, mean difference with 95% CIs, and relative differences in pension-qualifying incomes in mothers and fathers of children with type 1 diabetes (exposed) and their population-based matched controls (unexposed). Index years 1993-2014. Yearly incomes are reported in €100. 26

ESM Table 16. Mean absolute, mean difference with 95% CIs, and relative differences of pension-qualifying incomes in parents of children with type 1 diabetes (exposed) and the population-based matched parental controls (unexposed). Index years 1993-2004. Yearly incomes are reported in €100. 27

ESM Table 17. Post-hoc analysis. Mean absolute, mean difference with 95% CIs, and relative differences of pension-qualifying incomes, excluding the parental care allowance, in parents of children with type 1 diabetes (exposed) and the population-based matched parental controls (unexposed). Index years 1993-2014. Yearly incomes are reported in €100.............................. 28

ESM Table 18. Post-hoc analysis. Mean absolute, mean difference with 95% CIs, and relative differences of pension-qualifying incomes, excluding the parental care allowance, in parents of children with type 1 diabetes (exposed) and the population-based matched parental controls (unexposed). Index years 1993-2004. Yearly incomes are reported in €100.............................. 29

ESM Figure 1. Post hoc analysis. Yearly effect estimates from the difference-in-differences analysis (mean differences and 95% CIs) of work-related incomes in a) mothers, and b) fathers of children with type 1 diabetes by the number of children (0-17 years) residing in the parental household the year preceding the index year........ 31

ESM Figure 2. Yearly effect estimates from the difference-in-differences analysis (mean differences and 95% CIs) of pension-qualifying incomes in mothers (a) and fathers (b) of children with type 1 diabetes, with and without parental care allowance. Index years 1993-2014. Incomes are reported in €100. 32

ESM Figure 3. Sensitivity analysis. Top panels: Yearly effect estimates from the difference-in-differences analysis (mean differences and 95% CIs) of work-related incomes in mothers (a) and fathers (b) of children with type 1 diabetes. Bottom panels: Yearly effect estimates from the difference-in-differences analysis (mean differences and 95% CIs) of pension-qualifying incomes in mothers (c) and fathers (d) of children with type 1 diabetes, with and without parental care allowance. Index years 1997-2014. Incomes are reported in €100. 33

ESM Table 1. Baseline characteristics of mothers and fathers of children with type 1 diabetes (exposed) and their population-based controls (unexposed). Parental type 1 diabetes was assessed at the index date. Index years 1993-2014.

	Mothers Exposed	Mothers Unexposed	Fathers Exposed	Fathers Unexposed
N	13 182	256 187	12 906	250 329
Age at index date, median (IQR), yrs	38.0 (34.0:43.0)	38.0 (34.0:43.0)	41.0 (36.0:46.0)	41.0 (36.0:46.0)
Index year, N (%)				
1993-2000	2 717 (20.6)	52 855 (20.6)	2 657 (20.6)	51 494 (20.6)
2001-2007	4 998 (37.9)	97 174 (37.9)	4 903 (38.0)	95 075 (38.0)
2008-2014	5 467 (41.5)	106 158 (41.4)	5 346 (41.4)	103 760 (41.4)
Type 1 diabetes, N (%)	465 (3.5)	1 452 (0.6)	671 (5.2)	2 247 (0.9)
Country of birth, N (%)*				
Sweden	11 690 (88.7)	207 933 (81.2)	11 473 (88.9)	203 663 (81.4)
Europe (not Sweden)	760 (5.8)	21 984 (8.6)	674 (5.2)	21 907 (8.8)
Other	732 (5.6)	26 265 (10.3)	759 (5.9)	24 751 (9.9)
Highest education, N (%)*				
Compulsory	1 414 (10.7)	31 228 (12.2)	1 842 (14.3)	39 087 (15.6)
Secondary	6 662 (50.5)	123 654 (48.3)	6 937 (53.8)	128 280 (51.2)
University	5 032 (38.2)	97 946 (38.2)	4 048 (31.4)	80 694 (32.2)
Marital status, N (%)*				
Married	7 157 (54.3)	145 478 (56.8)	7 151 (55.4)	145 166 (58.0)
Cohabiting	3 445 (26.1)	60 198 (23.5)	3 396 (26.3)	59 135 (23.6)
Single	2 580 (19.6)	50 508 (19.7)	2 359 (18.3)	46 028 (18.4)
Number of children in the household, N (%)				
0	358 (2.7)	6 763 (2.6)	1 873 (14.5)	37 589 (15.0)
1	2 805 (21.3)	48 761 (19.0)	2 314 (17.9)	40 236 (16.1)
2	6 546 (49.7)	123 983 (48.4)	5 740 (44.5)	107 337 (42.9)
≥3	3 473 (26.3)	76 680 (29.9)	2 979 (23.1)	65 167 (26.0)
Region of residence, N (%)				
Götaland	6 457 (49.0)	123 313 (48.1)	6 316 (48.9)	120 504 (48.1)
Svealand	4 969 (37.7)	101 922 (39.8)	4 848 (37.6)	99 208 (39.6)
S Norrland	1 021 (7.7)	17 101 (6.7)	1 003 (7.8)	17 039 (6.8)
N Norrland	735 (5.6)	13 851 (5.4)	739 (5.7)	13 578 (5.4)
Population density, median (IQR)	75.7 (27.2:224.2)	82.1 (30.4:373.1)	75.7 (27.2:227.5)	81.8 (30.2:370.7)

* Column percentages do not add to 100 due to missing data. Missingness was <1.3% for education level, <0.01% for country of birth, and marital status.

ESM Table 2. Baseline characteristics of the children diagnosed with type 1 diabetes and the children of the population-based matched parental controls. Index years 1993-2014.

	**Children with type 1 diabetes**	**Children of population-based parental controls**
N	13 358	506 516
Boy, No (%)	7 191 (53.8)	260 276 (51.4)
Age at index date, median (IQR), yrs	9.0 (5.4:12.4)	8.9 (5.3:12.4)
Country of birth, No (%)		
Sweden	13 010 (97.4)	485 108 (95.8)
Europe (not Sweden)	178 (1.3)	9 704 (1.9)
Other	170 (1.3)	11 699 (2.3)

ESM Table 3. Mean absolute, mean differences and 95% CIs, and relative differences in work-related incomes in mothers and fathers of children with type 1 diabetes (exposed) and their population-based matched controls (unexposed). Index years 1993-2014. Yearly incomes are reported in €100.

	**Mothers**				**Fathers**			
**Year (t)**	**Exposed Mean (SD)**	**Unexposed Mean (SD)**	**Mean difference (95% CI)**	**Relative difference (%)**	**Exposed Mean (SD)**	**Unexposed Mean (SD)**	**Mean difference (95% CI)**	**Relative difference (%)**
-3	179 (153)	174 (159)	-0.2 (-2.2, 1.8)	-0.1	328 (249)	320 (287)	0.9 (-2.0, 3.8)	0.3
-2	190 (160)	184 (166)	0.3 (-1.3, 1.9)	0.2	339 (264)	330 (293)	0.8 (-1.5, 3.1)	0.2
-1	202 (170)	196 (173)	0	0	349 (275)	341 (306)	0	0
Index	202 (169)	213 (183)	-17.3 (-18.8, -15.8)	-7.9	352 (291)	354 (317)	-9.4 (-11.9, -7.0)	-2.6
+1	219 (177)	229 (186)	-15.4 (-17.2, -13.6)	-6.6	368 (295)	366 (325)	-6.0 (-8.9, -3.2)	-1.6
+2	235 (184)	244 (194)	-14.5 (-16.6, -12.5)	-5.8	378 (292)	378 (340)	-7.4 (-10.7, -4.1)	-1.9
+3	250 (197)	258 (200)	-13.7 (-16.0, -11.4)	-5.2	389 (307)	389 (347)	-6.8 (-10.4, -3.3)	-1.7
+4	263 (203)	271 (206)	-14.5 (-16.9, -12.1)	-5.2	400 (311)	399 (350)	-6.0 (-9.8, -2.3)	-1.5
+5	276 (213)	284 (215)	-13.6 (-16.2, -11.0)	-4.7	410 (322)	408 (354)	-5.9 (-9.9, -1.9)	-1.4
+6	289 (228)	296 (219)	-12.9 (-15.8, -10.0)	-4.3	418 (335)	416 (362)	-5.6 (-9.9, -1.4)	-1.3
+7	300 (227)	307 (224)	-12.3 (-15.3, -9.4)	-3.9	427 (348)	423 (395)	-4.1 (-8.7, 0.5)	-0.9

ESM Table 4. P-values for the interaction of average treatment effect across 7 years in a) mothers versus fathers, and b) in subgroup analyses.

a)

	p_{int}
Work-related incomes	<.001
Pension-qualifying income	<.001
Pension-qualifying income excluding parental care allowance	<.001

b)

	Mothers p_{int}	Fathers p_{int}
Age of child at index	<.001	0.005
Sex	0.461	0.727
Index year	0.075	0.643
Education level	0.733	0.947
Marital status	0.010	0.462
Country of birth	0.755	0.250

ESM Table 5. Sensitivity analysis excluding all parents diagnosed with type 1 diabetes at index date. Mean differences and 95% CIs of work-related incomes in parents of children with type 1 diabetes (exposed) and the population-based matched parental controls (unexposed). Index years 1993-2014. Yearly incomes are reported in €100.

	**Mean difference (95% CI)**	
**Year (t)**	**Mothers**	**Fathers**
-3	-0.4 (-2.4, 1.7)	1.1 (-1.9, 4.1)
-2	0.2 (-1.3, 1.8)	0.8 (-1.5, 3.2)
-1	0	0
Index	-17.3 (-18.8, -15.8)	-9.8 (-12.3, -7.2)
+1	-15.5 (-17.4, -13.6)	-6.1 (-9.1, -3.1)
+2	-14.5 (-16.6, -12.4)	-7.5 (-11.0, -4.1)
+3	-13.5 (-15.9, -11.2)	-6.7 (-10.4, -3.0)
+4	-14.5 (-16.9, -12.0)	-5.8 (-9.6, -1.9)
+5	-13.4 (-16.1, -10.7)	-5.4 (-9.5, -1.4)
+6	-12.7 (-15.6, -9.7)	-5.1 (-9.5, -0.7)
+7	-12.1 (-15.1, -9.1)	-3.7 (-8.5, 1.0)

ESM Table 6. Sensitivity analysis censoring exposed parents if a second child is diagnosed with type 1 diabetes. Mean differences and 95% CIs of work-related incomes in parents of children with type 1 diabetes (exposed) and the population-based matched parental controls (unexposed). Index years 1993-2014. Yearly incomes are reported in €100.

	**Mean difference (95% CI)**	
**Year (t)**	**Mothers**	**Fathers**
-3	-0.2 (-2.2, 1.8)	1.1 (-1.9, 4.0)
-2	0.2 (-1.4, 1.8)	0.8 (-1.5, 3.2)
-1	0	0
Index	-17.5 (-19.0, -16.0)	-9.4 (-11.9, -6.9)
+1	-15.4 (-17.3, -13.6)	-6.0 (-9.0, -3.1)
+2	-14.8 (-16.9, -12.7)	-7.4 (-10.8, -3.9)
+3	-13.9 (-16.2, -11.5)	-6.9 (-10.6, -3.2)
+4	-14.6 (-17.1, -12.2)	-6.3 (-10.1, -2.4)
+5	-13.7 (-16.4, -11.1)	-6.2 (-10.3, -2.1)
+6	-13.3 (-16.3, -10.4)	-6.0 (-10.4, -1.6)
+7	-12.6 (-15.5, -9.6)	-4.8 (-9.5, -0.1)

ESM Table 7. Subanalyses across child characteristics and calendar periods. Mean differences and 95% CIs of work-related incomes in mothers (a) and fathers (b) of children with type 1 diabetes (exposed) and the population-based matched parental controls (unexposed). Index years 1993-2014. Yearly incomes are reported in €100.

a. Mothers

	Mean difference (95% CI)							
	Age of child at index			Sex		Index year		
Year (t)	0.5-6	7-12	13-17	Girls	Boys	1993-2000	2001-2007	2008-2014
-3	0.1 (-3.7, 4.0)	-0.2 (-3.0, 2.6)	-0.7 (-4.4, 2.9)	-1.5 (-4.4, 1.4)	0.9 (-1.8, 3.6)	-0.4 (-4.3, 3.5)	-2.2 (-5.2, 0.9)	1.6 (-1.7, 5.0)
-2	-0.2 (-3.3, 3.0)	1.4 (-0.6, 3.5)	-1.1 (-4.1, 1.9)	-1.0 (-3.3, 1.3)	1.4 (-0.7, 3.6)	1.5 (-1.4, 4.3)	-0.9 (-3.3, 1.6)	0.8 (-1.9, 3.5)
-1	0	0	0	0	0	0	0	0
Index	-18.1 (-20.8, -15.3)	-18.6 (-20.7, -16.5)	-13.3 (-16.2, -10.3)	-19.0 (-21.1, -16.8)	-15.9 (-17.9, -13.8)	-14.1 (-16.9, -11.3)	-17.7 (-19.9, -15.5)	-18.5 (-21.1, -15.9)
+1	-20.7 (-24.1, -17.4)	-15.4 (-18.1, -12.6)	-6.3 (-9.9, -2.8)	-17.6 (-20.4, -14.8)	-13.5 (-16.0, -11.0)	-17.0 (-20.6, -13.5)	-13.6 (-16.4, -10.8)	-16.2 (-19.4, -13.1)
+2	-20.2 (-23.8, -16.6)	-14.8 (-17.8, -11.7)	-4.5 (-8.6, -0.3)	-16.6 (-19.6, -13.6)	-12.8 (-15.5, -10.0)	-16.3 (-20.1, -12.4)	-12.2 (-15.3, -9.1)	-15.8 (-19.3, -12.3)
+3	-20.0 (-23.9, -16.2)	-15.5 (-18.9, -12.2)	0.7 (-4.5, 5.9)	-15.4 (-18.7, -12.1)	-12.3 (-15.4, -9.1)	-16.8 (-21.1, -12.4)	-12.3 (-15.6, -8.9)	-13.5 (-17.6, -9.5)
+4	-21.2 (-25.2, -17.1)	-16.8 (-20.3, -13.2)	1.1 (-4.4, 6.7)	-16.9 (-20.3, -13.4)	-12.5 (-15.9, -9.1)	-16.2 (-20.8, -11.7)	-13.7 (-17.2, -10.2)	-14.4 (-18.7, -10.1)
+5	-20.6 (-24.8, -16.4)	-14.4 (-18.3, -10.6)	-0.3 (-6.3, 5.8)	-14.8 (-18.5, -11.0)	-12.6 (-16.3, -9.0)	-15.7 (-20.8, -10.6)	-12.2 (-16.0, -8.5)	-13.9 (-18.5, -9.2)
+6	-21.1 (-25.4, -16.7)	-12.4 (-16.8, -7.9)	-0.3 (-7.0, 6.5)	-13.7 (-18.1, -9.4)	-12.2 (-16.0, -8.3)	-16.1 (-21.2, -10.9)	-11.4 (-15.2, -7.5)	-12.7 (-18.1, -7.4)
+7	-22.4 (-26.9, -17.8)	-10.9 (-15.1, -6.7)	1.6 (-5.7, 8.9)	-12.6 (-16.7, -8.4)	-12.2 (-16.2, -8.1)	-17.1 (-22.5, -11.7)	-10.1 (-14.1, -6.0)	-12.0 (-17.4, -6.7)

b. Fathers

	Mean difference (95% CI)							
	**Age of child at index**			**Sex**		**Index year**		
**Year (t)**	**0.5-6**	**7-12**	**13-17**	**Girls**	**Boys**	**1993-2000**	**2001-2007**	**2008-2014**
-3	1.5 (-3.2, 6.2)	-1.5 (-6.3, 3.2)	5.0 (-0.7, 10.8)	0.8 (-3.2, 4.7)	1.1 (-3.2, 5.3)	2.1 (-2.4, 6.6)	-0.0 (-4.8, 4.8)	1.2 (-3.8, 6.2)
-2	0.9 (-3.0, 4.9)	-1.6 (-4.8, 1.6)	5.5 (0.1, 10.8)	1.1 (-2.4, 4.6)	0.5 (-2.5, 3.5)	1.3 (-2.8, 5.3)	0.1 (-3.1, 3.3)	1.2 (-3.0, 5.4)
-1	0	0	0	0	0	0	0	0
Index	-17.5 (-20.9, -14.1)	-5.8 (-10.0, -1.5)	-2.8 (-7.9, 2.3)	-8.7 (-11.9, -5.5)	-10.0 (-13.7, -6.4)	-8.2 (-13.1, -3.2)	-8.4 (-11.9, -4.9)	-11.0 (-15.3, -6.6)
+1	-11.6 (-15.8, -7.4)	-4.7 (-9.2, -0.1)	1.0 (-5.9, 7.8)	-5.7 (-9.5, -1.9)	-6.3 (-10.5, -2.2)	-4.1 (-9.8, 1.7)	-6.7 (-10.9, -2.5)	-6.4 (-11.4, -1.5)
+2	-9.3 (-14.0, -4.6)	-7.1 (-12.8, -1.5)	-4.5 (-11.7, 2.7)	-7.6 (-11.8, -3.5)	-7.2 (-12.2, -2.1)	-8.7 (-14.1, -3.2)	-8.2 (-14.2, -2.3)	-6.0 (-11.2, -0.7)
+3	-10.3 (-15.6, -5.0)	-7.3 (-13.4, -1.1)	0.1 (-7.0, 7.2)	-9.2 (-13.6, -4.7)	-4.8 (-10.2, 0.6)	-9.7 (-15.6, -3.8)	-8.8 (-15.0, -2.6)	-3.7 (-9.4, 2.1)
+4	-11.0 (-16.5, -5.5)	-5.5 (-11.9, 0.9)	1.5 (-6.0, 9.1)	-4.7 (-9.8, 0.3)	-7.1 (-12.5, -1.7)	-9.0 (-15.7, -2.3)	-6.3 (-12.9, 0.3)	-4.3 (-10.2, 1.5)
+5	-12.6 (-18.7, -6.5)	-4.3 (-10.7, 2.1)	2.5 (-6.1, 11.0)	-3.9 (-9.5, 1.7)	-7.7 (-13.3, -2.1)	-8.8 (-16.0, -1.7)	-7.0 (-13.5, -0.4)	-3.5 (-10.0, 3.1)
+6	-12.7 (-19.0, -6.4)	-3.4 (-10.3, 3.5)	2.3 (-7.7, 12.2)	-4.7 (-10.7, 1.2)	-6.4 (-12.5, -0.3)	-8.5 (-16.2, -0.8)	-7.6 (-14.3, -0.9)	-2.4 (-9.7, 5.0)
+7	-10.1 (-17.1, -3.1)	-2.1 (-9.4, 5.2)	2.4 (-7.9, 12.7)	-3.8 (-10.3, 2.6)	-4.2 (-10.7, 2.3)	-5.5 (-13.9, 3.0)	-6.4 (-13.7, 0.9)	-1.3 (-9.0, 6.5)

ESM Table 8. Baseline characteristics of mothers (a) and fathers (b) of children with type 1 diabetes and their population-based controls, categorized by age of child at index. Parental type 1 diabetes was assessed at the index date. Index years 1993-2014.

a) Mothers

	Age of child at index					
	0.5-6		7-12		13-17	
	Exposed	Unexposed	Exposed	Unexposed	Exposed	Unexposed
N	4 767	93 012	5 589	108 370	2 826	54 805
Age at index date, median (IQR), yrs	34.0 (30.0:38.0)	34.0 (30.0:38.0)	39.0 (36.0:43.0)	39.0 (36.0:43.0)	44.0 (40.0:47.0)	44.0 (40.0:47.0)
Index year, N (%)						
1993-2000	1 572 (33.0)	30 743 (33.1)	1 119 (20.0)	21 579 (19.9)	26 (0.9)	533 (1.0)
2001-2007	1 450 (30.4)	28 220 (30.3)	2 288 (40.9)	44 602 (41.2)	1 260 (44.6)	24 352 (44.4)
2008-2014	1 745 (36.6)	34 049 (36.6)	2 182 (39.0)	42 189 (38.9)	1 540 (54.5)	29 920 (54.6)
Type 1 diabetes, N (%)	160 (3.4)	463 (0.5)	185 (3.3)	607 (0.6)	120 (4.2)	382 (0.7)
Country of birth, N (%)*						
Sweden	4 240 (88.9)	75 373 (81.0)	4 950 (88.6)	88 254 (81.4)	2 500 (88.5)	44 306 (80.8)
Europe (not Sweden)	252 (5.3)	7 620 (8.2)	334 (6.0)	9 333 (8.6)	174 (6.2)	5 031 (9.2)
Other	275 (5.8)	10 015 (10.8)	305 (5.5)	10 782 (9.9)	152 (5.4)	5 468 (10.0)
Highest education, N (%)*						
Compulsory	540 (11.3)	11 489 (12.4)	549 (9.8)	13 094 (12.1)	325 (11.5)	6 645 (12.1)
Secondary	2 335 (49.0)	43 619 (46.9)	2 894 (51.8)	52 816 (48.7)	1 433 (50.7)	27 219 (49.7)
University	1 858 (39.0)	36 283 (39.0)	2 122 (38.0)	41 217 (38.0)	1 052 (37.2)	20 446 (37.3)
Marital status, N (%)*						
Married	2 464 (51.7)	50 646 (54.5)	3 118 (55.8)	63 165 (58.3)	1 575 (55.7)	31 667 (57.8)
Cohabiting	1 700 (35.7)	29 870 (32.1)	1 270 (22.7)	21 826 (20.1)	475 (16.8)	8 502 (15.5)
Single	603 (12.6)	12 494 (13.4)	1 201 (21.5)	23 378 (21.6)	776 (27.5)	14 636 (26.7)
Number of children in the household, N (%)						
0	58 (1.2)	1 271 (1.4)	159 (2.8)	3 087 (2.8)	141 (5.0)	2 405 (4.4)
1	1 179 (24.7)	20 648 (22.2)	849 (15.2)	13 809 (12.7)	777 (27.5)	14 304 (26.1)
2	2 417 (50.7)	46 333 (49.8)	2 906 (52.0)	53 869 (49.7)	1 223 (43.3)	23 781 (43.4)
>=3	1 113 (23.3)	24 760 (26.6)	1 675 (30.0)	37 605 (34.7)	685 (24.2)	14 315 (26.1)
Region of residence, N (%)						
Götaland	2 330 (48.9)	44 437 (47.8)	2 741 (49.0)	52 253 (48.2)	1 386 (49.0)	26 623 (48.6)

	Age of child at index					
	0.5-6		7-12		13-17	
	**Exposed**	**Unexposed**	**Exposed**	**Unexposed**	**Exposed**	**Unexposed**
Svealand	1 797 (37.7)	37 666 (40.5)	2 094 (37.5)	42 953 (39.6)	1 078 (38.1)	21 303 (38.9)
S Norrland	365 (7.7)	5 939 (6.4)	448 (8.0)	7 410 (6.8)	208 (7.4)	3 752 (6.8)
N Norrland	275 (5.8)	4 970 (5.3)	306 (5.5)	5 754 (5.3)	154 (5.4)	3 127 (5.7)
Population density, median (IQR)	76.8 (27.9:256.8)	83.0 (32.1:449.5)	76.2 (27.1:191.8)	81.6 (29.8:356.4)	70.3 (26.9:165.3)	81.3 (29.3:295.6)

* Column percentages do not add to 100 due to missing data. Missingness was <1.8% for education level, <0.01% for country of birth, and marital status.

b) Fathers

	Age of child at index					
	0.5-6		7-12		13-17	
	Exposed	Unexposed	Exposed	Unexposed	Exposed	Unexposed
N	4 705	91 620	5 474	105 990	2 727	52 719
Age at index date, median (IQR), yrs	36.0 (32.0:40.0)	36.0 (32.0:40.0)	42.0 (38.0:46.0)	42.0 (38.0:46.0)	46.0 (42.0:50.0)	46.0 (43.0:50.0)
Index year, N (%)						
1993-2000	1 544 (32.8)	30 049 (32.8)	1 088 (19.9)	20 918 (19.7)	25 (0.9)	527 (1.0)
2001-2007	1 433 (30.5)	27 872 (30.4)	2 251 (41.1)	43 852 (41.4)	1 219 (44.7)	23 351 (44.3)
2008-2014	1 728 (36.7)	33 699 (36.8)	2 135 (39.0)	41 220 (38.9)	1 483 (54.4)	28 841 (54.7)
Type 1 diabetes, N (%)	264 (5.6)	679 (0.7)	269 (4.9)	968 (0.9)	138 (5.1)	600 (1.1)
Country of birth, N (%)*						
Sweden	4 190 (89.1)	74 320 (81.1)	4 839 (88.4)	86 397 (81.5)	2 444 (89.6)	42 946 (81.5)
Europe (not Sweden)	239 (5.1)	7 830 (8.5)	296 (5.4)	9 319 (8.8)	139 (5.1)	4 758 (9.0)
Other	276 (5.9)	9 465 (10.3)	339 (6.2)	10 273 (9.7)	144 (5.3)	5 013 (9.5)
Highest education, N (%)*						
Compulsory	598 (12.7)	13 393 (14.6)	793 (14.5)	16 846 (15.9)	451 (16.5)	8 848 (16.8)
Secondary	2 556 (54.3)	46 446 (50.7)	2 939 (53.7)	54 407 (51.3)	1 442 (52.9)	27 427 (52.0)
University	1 523 (32.4)	30 639 (33.4)	1 707 (31.2)	33 917 (32.0)	818 (30.0)	16 138 (30.6)
Marital status, N (%)*						
Married	2 483 (52.8)	50 858 (55.5)	3 134 (57.3)	63 191 (59.6)	1 534 (56.3)	31 117 (59.0)
Cohabiting	1 697 (36.1)	29 548 (32.3)	1 233 (22.5)	21 309 (20.1)	466 (17.1)	8 278 (15.7)
Single	525 (11.2)	11 214 (12.2)	1 107 (20.2)	21 490 (20.3)	727 (26.7)	13 324 (25.3)
Number of children in the household, N (%)						
0	471 (10.0)	9 944 (10.9)	857 (15.7)	17 282 (16.3)	545 (20.0)	10 363 (19.7)
1	1 005 (21.4)	17 676 (19.3)	682 (12.5)	10 919 (10.3)	627 (23.0)	11 641 (22.1)
2	2 209 (47.0)	42 037 (45.9)	2 524 (46.1)	46 039 (43.4)	1 007 (36.9)	19 261 (36.5)
>=3	1 020 (21.7)	21 963 (24.0)	1 411 (25.8)	31 750 (30.0)	548 (20.1)	11 454 (21.7)
Region of residence, N (%)						
Götaland	2 308 (49.1)	43 810 (47.8)	2 671 (48.8)	51 042 (48.2)	1 337 (49.0)	25 652 (48.7)
Svealand	1 757 (37.3)	36 935 (40.3)	2 057 (37.6)	41 831 (39.5)	1 034 (37.9)	20 442 (38.8)
S Norrland	363 (7.7)	6 034 (6.6)	438 (8.0)	7 268 (6.9)	202 (7.4)	3 737 (7.1)

	Age of child at index					
	0.5-6		7-12		13-17	
	Exposed	Unexposed	Exposed	Unexposed	Exposed	Unexposed
N Norrland	277 (5.9)	4 841 (5.3)	308 (5.6)	5 849 (5.5)	154 (5.6)	2 888 (5.5)
Population density, median (IQR)	76.7 (28.9:252.6)	83.0 (31.9:451.1)	76.1 (27.2:225.4)	80.8 (29.7:350.0)	70.5 (26.9:171.0)	80.6 (29.0:267.5)

* Column percentages do not add to 100 due to missing data. Missingness was <1.3% for education level, <0.01% for country of birth, and marital status.

ESM Table 9. Subanalyses across parental sociodemographic characteristics. Mean differences and 95% CIs of work-related incomes in mothers (a) and fathers (b) of children with type 1 diabetes (exposed) and the population-based matched parental controls (unexposed). Index years 1993-2014. Yearly incomes are reported in €100.

a. Mothers

	Mean difference (95% CI)							
	Education level			Marital status		Country of birth		
Year (t)	Compulsory	Secondary	University	Single	Cohabitating	Sweden	Europe (not Swe)	Others
-3	1.6 (-3.0, 6.1)	0.3 (-2.2, 2.8)	-1.1 (-4.9, 2.6)	-4.4 (-8.7, -0.1)	0.8 (-1.4, 3.0)	0.6 (-1.6, 2.7)	-8.4 (-16.6, -0.2)	1.2 (-5.6, 8.1)
-2	-0.8 (-4.2, 2.6)	0.8 (-1.0, 2.7)	0.1 (-3.0, 3.2)	-1.3 (-4.5, 1.8)	0.7 (-1.1, 2.5)	0.8 (-0.9, 2.5)	-4.3 (-10.7, 2.0)	0.4 (-6.2, 6.9)
-1	0	0	0	0	0	0	0	0
Index	-11.1 (-14.4, -7.7)	-16.6 (-18.4, -14.8)	-20.4 (-23.3, -17.5)	-14.1 (-17.5, -10.8)	-18.1 (-19.7, -16.4)	-17.7 (-19.3, -16.2)	-16.9 (-22.9, -10.8)	-14.9 (-20.5, -9.4)
+1	-11.8 (-16.2, -7.5)	-14.1 (-16.4, -11.9)	-18.7 (-22.2, -15.2)	-13.3 (-17.4, -9.3)	-15.9 (-18.0, -13.8)	-15.9 (-17.8, -13.9)	-19.9 (-27.2, -12.6)	-10.2 (-18.7, -1.8)
+2	-11.8 (-16.7, -7.0)	-14.1 (-16.6, -11.5)	-16.7 (-20.6, -12.8)	-9.8 (-14.7, -4.9)	-15.7 (-17.9, -13.5)	-15.0 (-17.2, -12.9)	-15.2 (-23.3, -7.0)	-13.9 (-23.2, -4.6)
+3	-11.5 (-17.0, -6.0)	-14.4 (-17.1, -11.7)	-14.3 (-18.8, -9.9)	-8.8 (-14.8, -2.8)	-14.9 (-17.3, -12.5)	-14.3 (-16.8, -11.9)	-14.7 (-23.9, -5.6)	-11.9 (-21.8, -1.9)
+4	-13.0 (-18.8, -7.3)	-14.2 (-17.1, -11.4)	-16.3 (-21.0, -11.6)	-11.6 (-17.9, -5.2)	-15.3 (-17.8, -12.7)	-15.0 (-17.6, -12.4)	-16.7 (-26.3, -7.1)	-14.4 (-24.8, -4.1)
+5	-13.0 (-19.0, -7.0)	-14.4 (-17.4, -11.3)	-13.8 (-18.9, -8.7)	-9.4 (-16.2, -2.5)	-14.7 (-17.4, -11.9)	-14.1 (-16.9, -11.4)	-16.3 (-26.4, -6.2)	-14.3 (-24.8, -3.8)
+6	-13.0 (-19.6, -6.4)	-13.3 (-16.4, -10.1)	-13.1 (-18.9, -7.2)	-9.3 (-16.7, -1.9)	-13.8 (-16.9, -10.7)	-13.2 (-16.3, -10.1)	-17.8 (-28.6, -7.0)	-14.7 (-26.0, -3.4)
+7	-12.3 (-19.0, -5.6)	-13.0 (-16.3, -9.6)	-12.5 (-18.2, -6.7)	-7.0 (-15.0, 1.0)	-13.7 (-16.7, -10.6)	-13.1 (-16.2, -10.0)	-14.1 (-25.0, -3.3)	-10.8 (-22.6, 1.0)

b. Fathers

	Mean difference (95% CI)							
	Education level			Marital status		Country of birth		
Year (t)	Compulsory	Secondary	University	Single	Cohabitating	Sweden	Europe (not Swe)	Others
-3	-0.2 (-4.8, 4.5)	-0.7 (-3.6, 2.3)	5.4 (-1.8, 12.7)	-0.5 (-5.9, 5.0)	1.3 (-2.0, 4.7)	1.0 (-2.1, 4.1)	5.7 (-7.3, 18.7)	0.4 (-11.9, 12.8)
-2	1.1 (-2.5, 4.7)	0.3 (-2.2, 2.8)	2.2 (-3.3, 7.6)	3.3 (-0.8, 7.4)	0.3 (-2.3, 2.9)	0.8 (-1.6, 3.2)	1.2 (-8.2, 10.6)	2.9 (-6.8, 12.6)
-1	0	0	0	0	0	0	0	0
Index	-3.6 (-7.3, 0.0)	-12.4 (-14.9, -9.9)	-5.6 (-11.7, 0.4)	-5.4 (-9.6, -1.2)	-10.3 (-13.2, -7.5)	-9.7 (-12.1, -7.4)	-0.1 (-19.4, 19.2)	-13.2 (-27.4, 1.0)
+1	-6.4 (-11.5, -1.4)	-8.3 (-11.3, -5.4)	-0.3 (-7.1, 6.4)	-3.4 (-9.6, 2.7)	-6.6 (-9.8, -3.4)	-5.6 (-8.5, -2.6)	-1.5 (-11.4, 8.3)	-19.5 (-36.9, -2.0)
+2	-7.0 (-12.7, -1.3)	-6.1 (-9.4, -2.9)	-8.7 (-17.2, -0.3)	-6.9 (-13.4, -0.4)	-7.5 (-11.3, -3.7)	-7.2 (-10.7, -3.6)	-8.8 (-20.9, 3.4)	-13.2 (-27.6, 1.3)
+3	-5.9 (-12.0, 0.2)	-6.2 (-9.8, -2.5)	-7.3 (-16.3, 1.6)	-3.9 (-11.1, 3.3)	-7.5 (-11.6, -3.4)	-6.4 (-10.3, -2.5)	-11.3 (-23.7, 1.2)	-15.1 (-27.5, -2.7)
+4	-5.0 (-12.1, 2.0)	-5.9 (-9.7, -2.1)	-5.7 (-15.0, 3.5)	-0.9 (-8.5, 6.8)	-7.2 (-11.4, -3.0)	-5.8 (-9.9, -1.8)	-1.7 (-15.8, 12.4)	-17.7 (-30.9, -4.4)
+5	-7.8 (-15.0, -0.6)	-4.0 (-8.1, -0.0)	-7.4 (-17.2, 2.5)	-1.6 (-10.2, 6.9)	-6.8 (-11.3, -2.4)	-5.5 (-9.8, -1.2)	-6.6 (-20.1, 6.9)	-18.2 (-31.6, -4.8)
+6	-7.7 (-15.5, 0.1)	-5.0 (-9.4, -0.7)	-4.8 (-15.5, 5.8)	-5.2 (-14.1, 3.6)	-5.7 (-10.5, -0.9)	-5.2 (-9.8, -0.6)	-15.7 (-31.7, 0.3)	-12.7 (-27.0, 1.5)
+7	-6.6 (-15.1, 1.8)	-3.7 (-8.1, 0.8)	-2.9 (-14.5, 8.7)	-2.8 (-11.8, 6.1)	-4.4 (-9.6, 0.9)	-4.6 (-9.6, 0.3)	-3.9 (-21.9, 14.1)	-8.6 (-23.5, 6.2)

ESM Table 10. Post hoc analysis. Mean absolute, mean differences and 95% CIs, and relative differences in work-related incomes in mothers and fathers of children with type 1 diabetes (exposed) and their population-based matched controls (unexposed), by the maternal proportion of familial disposable income the year preceding the index year. Index years 1993-2014. Yearly incomes are reported in €100.

	Maternal proportion of familial disposable income the year preceding the index year							
	<50%				≥50%			
Year (t)	Exposed Mean (SD)	Unexposed Mean (SD)	Mean difference (95% CI)	Relative difference (%)	Exposed Mean (SD)	Unexposed Mean (SD)	Mean difference (95% CI)	Relative difference (%)
-3	170 (135)	166 (142)	1.1 (-1.3, 3.6)	0.7	224 (196)	216 (201)	-2.8 (-7.9, 2.3)	-1.2
-2	177 (140)	173 (144)	1.2 (-0.8, 3.1)	0.7	243 (203)	236 (216)	-2.8 (-6.9, 1.4)	-1.1
-1	184 (142)	181 (147)	0	0	277 (223)	265 (226)	0	0
Index	188 (142)	202 (155)	-17.5 (-19.3, -15.8)	-8.5	263 (219)	273 (248)	-21.0 (-24.9, -17.1)	-7.4
+1	209 (153)	221 (162)	-15.6 (-17.9, -13.4)	-7.0	277 (227)	285 (243)	-18.7 (-23.7, -13.8)	-6.3
+2	225 (158)	237 (167)	-15.6 (-18.0, -13.2)	-6.5	294 (231)	300 (253)	-17.6 (-23.0, -12.1)	-5.6
+3	241 (166)	252 (174)	-14.1 (-16.7, -11.5)	-5.5	306 (247)	315 (262)	-19.2 (-25.3, -13.2)	-5.9
+4	256 (171)	267 (178)	-14.3 (-17.0, -11.5)	-5.3	319 (255)	328 (273)	-20.2 (-26.6, -13.9)	-6.0
+5	268 (176)	280 (184)	-15.3 (-18.3, -12.4)	-5.4	339 (276)	342 (297)	-13.6 (-20.6, -6.7)	-3.9
+6	281 (181)	292 (190)	-14.4 (-17.5, -11.3)	-4.9	352 (318)	355 (292)	-13.1 (-21.7, -4.4)	-3.6
+7	293 (185)	304 (198)	-13.8 (-17.1, -10.6)	-4.5	362 (292)	366 (293)	-14.3 (-22.0, -6.6)	-3.8

ESM Table 11. Post hoc analysis. Mean differences and 95% CIs of work-related incomes in parents of children with type 1 diabetes (exposed) and the population-based matched parental controls (unexposed), by the number of children registered in the parental household the year preceding the index year. Index years 1993-2014. Yearly incomes are reported in €100.

	Mothers		Fathers	
	Mean difference (95% CI)		Mean difference (95% CI)	
	Number of children in the household		Number of children in the household	
Year (t)	≤1	≥2	≤1	≥2
-3	1.0 (-3.3, 5.2)	-1.1 (-3.4, 1.1)	-0.2 (-4.7, 4.3)	1.3 (-2.4, 5.0)
-2	1.3 (-1.9, 4.4)	-0.2 (-2.0, 1.6)	1.4 (-2.3, 5.2)	0.4 (-2.4, 3.3)
-1	0	0	0	0
Index	-14.1 (-17.1, -11.0)	-18.0 (-19.7, -16.3)	-7.6 (-11.7, -3.5)	-10.2 (-13.3, -7.1)
+1	-10.8 (-14.5, -7.2)	-16.1 (-18.3, -14.0)	-5.0 (-10.1, 0.2)	-6.3 (-9.7, -2.9)
+2	-9.4 (-13.4, -5.4)	-15.4 (-17.8, -13.1)	-6.1 (-11.4, -0.8)	-7.7 (-11.9, -3.5)
+3	-8.8 (-13.2, -4.4)	-14.6 (-17.2, -11.9)	-2.6 (-8.5, 3.2)	-8.5 (-13.0, -4.0)
+4	-10.4 (-15.0, -5.9)	-15.1 (-17.9, -12.3)	-1.7 (-7.9, 4.4)	-7.7 (-12.3, -3.0)
+5	-9.6 (-14.5, -4.7)	-14.1 (-17.1, -11.0)	0.0 (-7.2, 7.2)	-8.2 (-12.9, -3.5)
+6	-7.0 (-12.1, -1.8)	-13.9 (-17.3, -10.5)	-2.2 (-9.1, 4.7)	-6.6 (-12.0, -1.3)
+7	-6.0 (-11.4, -0.6)	-13.4 (-16.8, -10.0)	-0.5 (-7.8, 6.8)	-5.1 (-10.8, 0.7)

ESM Table 12. Baseline characteristics of mothers and fathers of children with type 1 diabetes (exposed) and their population-based controls (unexposed). Parental type 1 diabetes was assessed at the index date. Index years 1993-2004.

		Mothers Exposed	Mothers Unexposed	Fathers Exposed	Fathers Unexposed
N		5 395	104 941	5 288	102 487
Age at index date, median (IQR), yrs		36.0 (32.0:41.0)	36.0 (32.0:41.0)	39.0 (34.0:44.0)	39.0 (34.0:44.0)
Index year, N (%)					
Type 1 diabetes, N (%)		162 (3.0)	479 (0.5)	208 (3.9)	752 (0.7)
Country of birth, N (%)*					
Sweden		4 861 (90.1)	88 154 (84.0)	4 780 (90.4)	85 728 (83.6)
Europe (not Sweden)		331 (6.1)	8 896 (8.5)	271 (5.1)	8 714 (8.5)
Other		203 (3.8)	7 891 (7.5)	237 (4.5)	8 043 (7.8)
Highest education, N (%)*					
Compulsory		716 (13.3)	15 273 (14.6)	923 (17.5)	18 889 (18.4)
Secondary		2 978 (55.2)	55 474 (52.9)	2 868 (54.2)	53 410 (52.1)
University		1 676 (31.1)	32 729 (31.2)	1 472 (27.8)	29 096 (28.4)
Marital status, N (%)*					
Married		3 022 (56.0)	60 169 (57.3)	3 031 (57.3)	60 140 (58.7)
Cohabiting		1 380 (25.6)	25 005 (23.8)	1 372 (25.9)	24 484 (23.9)
Single		993 (18.4)	19 766 (18.8)	885 (16.7)	17 863 (17.4)
Number of children in the household, N (%)					
0		838 (7.8)	17 206 (8.3)	838 (7.8)	17 206 (8.3)
1		1 080 (20.0)	18 263 (17.4)	909 (17.2)	15 002 (14.6)
2		2 710 (50.2)	50 914 (48.5)	2 349 (44.4)	43 338 (42.3)
>=3		1 499 (27.8)	33 831 (32.2)	1 298 (24.5)	28 874 (28.2)
Region of residence, N (%)					
Götaland		2 633 (48.8)	50 704 (48.3)	2 580 (48.8)	49 509 (48.3)
Svealand		2 035 (37.7)	40 807 (38.9)	1 979 (37.4)	39 935 (39.0)
S Norrland		402 (7.5)	7 312 (7.0)	405 (7.7)	7 241 (7.1)
N Norrland		325 (6.0)	6 118 (5.8)	324 (6.1)	5 802 (5.7)
Population density, median (IQR)		69.1 (26.2:152.3)	76.0 (28.0:271.3)	69.3 (26.2:152.3)	76.0 (28.6:321.0)

* Column percentages do not add to 100 due to missing data. Missingness was 1.7% for education level, <0.01% for country of birth, and marital status.

ESM Table 13. Baseline characteristics of the children diagnosed with type 1 diabetes and the children of the population-based matched parental controls. Index years 1993-2004.

	**Children with type 1 diabetes**	**Children of population-based parental controls**
N	5 461	207 428
Boy, No (%)	2 852 (52.2)	106 378 (51.3)
Age at index date, median (IQR), yrs	7.6 (4.6:10.6)	7.6 (4.6:10.6)
Country of birth, No (%)		
Sweden	5 370 (98.3)	200 849 (96.8)
Europe (not Sweden)	54 (1.0)	3 473 (1.7)
Other	37 (0.7)	3 106 (1.5)

ESM Table 14. Mean absolute, mean differences and 95% CIs, and relative differences in work-related incomes in mothers and fathers of children with type 1 diabetes (exposed) and the population-based matched parental controls (unexposed). Index years 1993-2004. Yearly incomes are reported in €100.

	Mothers				Fathers			
**Year (t)**	**Exposed Mean (SD)**	**Unexposed Mean (SD)**	**Mean difference (95% CI)**	**Relative difference (%)**	**Exposed Mean (SD)**	**Unexposed Mean (SD)**	**Mean difference (95% CI)**	**Relative difference (%)**
-3	137 (116)	134 (122)	-0.7 (-3.5, 2.1)	-0.5	279 (190)	271 (230)	1.6 (-2.7, 5.9)	0.6
-2	147 (125)	142 (129)	0.3 (-1.9, 2.4)	0.2	290 (230)	282 (240)	1.3 (-1.8, 4.3)	0.4
-1	157 (133)	152 (133)	0	0	301 (246)	293 (269)	0	0
Index	155 (130)	166 (139)	-15.7 (-17.8, -13.7)	-9.2	304 (278)	304 (268)	-7.4 (-10.8, -4.0)	-2.4
+1	169 (139)	180 (146)	-15.5 (-18.1, -12.9)	-8.4	320 (260)	316 (278)	-2.8 (-7.1, 1.6)	-0.9
+2	183 (144)	193 (152)	-14.7 (-17.5, -11.9)	-7.5	326 (247)	327 (279)	-7.6 (-13.4, -1.7)	-2.3
+3	193 (150)	205 (160)	-16.2 (-19.4, -13.1)	-7.7	337 (248)	339 (290)	-8.4 (-14.4, -2.4)	-2.4
+4	205 (154)	217 (167)	-17.1 (-20.4, -13.9)	-7.7	347 (265)	350 (294)	-8.1 (-14.4, -1.8)	-2.3
+5	219 (164)	230 (171)	-15.6 (-19.1, -12.0)	-6.6	357 (257)	359 (307)	-8.9 (-14.9, -3.0)	-2.4
+6	231 (167)	241 (174)	-14.6 (-18.2, -10.9)	-5.9	365 (262)	367 (312)	-9.1 (-15.4, -2.8)	-2.4
+7	242 (169)	252 (185)	-14.8 (-18.6, -11.0)	-5.8	373 (269)	375 (326)	-8.1 (-14.8, -1.4)	-2.1
+8	253 (178)	262 (182)	-14.1 (-18.2, -10.1)	-5.3	379 (282)	382 (329)	-9.2 (-16.1, -2.3)	-2.4
+9	262 (182)	273 (191)	-15.0 (-19.1, -10.9)	-5.4	389 (318)	388 (363)	-5.6 (-13.6, 2.3)	-1.4
+10	272 (186)	283 (193)	-15.0 (-19.3, -10.7)	-5.2	394 (310)	393 (361)	-5.7 (-13.6, 2.2)	-1.4
+11	284 (192)	293 (196)	-13.3 (-17.8, -8.8)	-4.5	400 (316)	399 (347)	-5.9 (-14.0, 2.1)	-1.5
+12	293 (199)	302 (202)	-12.5 (-17.2, -7.7)	-4.1	406 (333)	403 (341)	-4.4 (-13.0, 4.2)	-1.1
+13	303 (211)	310 (210)	-11.5 (-16.6, -6.3)	-3.6	407 (349)	406 (351)	-6.9 (-16.0, 2.2)	-1.7
+14	308 (216)	317 (214)	-12.7 (-18.1, -7.4)	-4.0	407 (361)	407 (354)	-7.4 (-17.1, 2.4)	-1.8
+15	315 (231)	322 (219)	-10.9 (-16.7, -5.1)	-3.3	405 (365)	407 (365)	-8.4 (-18.5, 1.6)	-2.0
+16	319 (234)	325 (224)	-10.5 (-16.5, -4.5)	-3.2	403 (366)	403 (364)	-7.2 (-17.3, 3.0)	-1.7
+17	321 (240)	327 (231)	-10.3 (-16.6, -4.1)	-3.1	397 (384)	399 (389)	-9.1 (-19.9, 1.7)	-2.2

ESM Table 15. Mean absolute, mean difference with 95% CIs, and relative differences in pension-qualifying incomes in mothers and fathers of children with type 1 diabetes (exposed) and their population-based matched controls (unexposed). Index years 1993-2014. Yearly incomes are reported in €100.

	Mothers				Fathers			
Year (t)	Exposed Mean (SD)	Unexposed Mean (SD)	Mean difference (95% CI)	Relative difference (%)	Exposed Mean (SD)	Unexposed Mean (SD)	Mean difference (95% CI)	Relative difference (%)
-3	224 (137)	217 (147)	-0.8 (-2.4, 0.7)	-0.4	351 (239)	343 (279)	1.1 (-1.7, 3.9)	0.3
-2	234 (145)	226 (153)	-0.4 (-1.6, 0.8)	-0.2	361 (255)	353 (284)	0.4 (-1.7, 2.6)	0.1
-1	244 (154)	235 (160)	0	0	372 (265)	364 (297)	0	0
Index	265 (155)	248 (170)	8.3 (7.0, 9.5)	3.2	384 (281)	375 (308)	0.2 (-2.2, 2.5)	0.0
+1	299 (159)	262 (173)	28.7 (27.1, 30.3)	10.6	398 (284)	387 (316)	3.1 (0.3, 5.8)	0.8
+2	301 (165)	275 (180)	17.6 (15.8, 19.4)	6.2	405 (281)	398 (331)	-1.2 (-4.4, 2.0)	-0.3
+3	312 (178)	288 (186)	15.2 (13.2, 17.3)	5.1	415 (295)	409 (338)	-1.6 (-5.1, 1.9)	-0.4
+4	321 (184)	301 (191)	11.5 (9.4, 13.7)	3.7	425 (299)	418 (340)	-1.2 (-4.8, 2.4)	-0.3
+5	330 (194)	313 (201)	9.2 (6.8, 11.5)	2.9	433 (310)	427 (344)	-1.6 (-5.4, 2.3)	-0.4
+6	339 (210)	324 (203)	6.7 (4.1, 9.4)	2.0	440 (324)	434 (352)	-1.9 (-6.1, 2.2)	-0.4
+7	347 (209)	334 (209)	4.5 (1.8, 7.2)	1.3	448 (336)	441 (385)	-1.1 (-5.5, 3.4)	-0.2

ESM Table 16. Mean absolute, mean difference with 95% CIs, and relative differences of pension-qualifying incomes in parents of children with type 1 diabetes (exposed) and the population-based matched parental controls (unexposed). Index years 1993-2004. Yearly incomes are reported in €100.

	Mothers				Fathers			
**Year (t)**	**Exposed Mean (SD)**	**Unexposed Mean (SD)**	**Mean difference (95% CI)**	**Relative difference (%)**	**Exposed Mean (SD)**	**Unexposed Mean (SD)**	**Mean difference (95% CI)**	**Relative difference (%)**
-3	184 (102)	178 (111)	-1.4 (-3.6, 0.7)	-0.8	301 (178)	293 (221)	1.2 (-3.0, 5.3)	0.4
-2	192 (110)	185 (117)	-0.6 (-2.2, 1.1)	-0.3	313 (220)	304 (231)	1.2 (-1.7, 4.1)	0.4
-1	201 (118)	193 (121)	0	0	323 (235)	316 (260)	0	0
Index	223 (118)	204 (127)	11.0 (9.3, 12.7)	5.2	336 (267)	327 (258)	1.7 (-1.6, 5.0)	0.5
+1	260 (122)	216 (133)	36.5 (34.3, 38.8)	16.3	350 (246)	338 (267)	5.4 (1.2, 9.6)	1.6
+2	259 (125)	228 (138)	23.0 (20.5, 25.4)	9.7	355 (232)	350 (268)	-1.6 (-7.3, 4.0)	-0.5
+3	266 (130)	240 (144)	18.8 (16.1, 21.6)	7.6	364 (233)	361 (278)	-3.4 (-9.2, 2.5)	-0.9
+4	273 (134)	251 (151)	14.8 (12.0, 17.7)	5.7	374 (251)	371 (282)	-3.1 (-9.2, 3.0)	-0.8
+5	283 (143)	262 (154)	13.2 (10.1, 16.3)	4.9	383 (241)	381 (295)	-4.2 (-10.0, 1.5)	-1.1
+6	290 (145)	273 (156)	9.8 (6.6, 13.1)	3.5	390 (246)	388 (300)	-5.3 (-11.4, 0.8)	-1.3
+7	295 (148)	282 (167)	6.0 (2.7, 9.4)	2.1	397 (253)	395 (315)	-4.7 (-11.2, 1.8)	-1.2
+8	302 (157)	291 (164)	3.6 (-0.0, 7.1)	1.2	402 (267)	401 (317)	-5.5 (-12.2, 1.1)	-1.4
+9	308 (160)	300 (173)	0.5 (-3.2, 4.1)	0.1	410 (306)	407 (352)	-3.4 (-11.2, 4.3)	-0.8
+10	315 (165)	310 (174)	-2.1 (-5.9, 1.7)	-0.7	415 (297)	412 (350)	-3.5 (-11.2, 4.2)	-0.8
+11	323 (172)	319 (178)	-3.4 (-7.4, 0.7)	-1.0	420 (304)	418 (336)	-4.5 (-12.4, 3.4)	-1.1
+12	329 (179)	328 (183)	-5.7 (-10.0, -1.4)	-1.7	425 (321)	422 (329)	-2.9 (-11.3, 5.6)	-0.7
+13	337 (191)	336 (192)	-6.4 (-11.1, -1.8)	-1.9	426 (338)	424 (340)	-5.4 (-14.3, 3.5)	-1.2
+14	340 (197)	343 (196)	-9.6 (-14.4, -4.7)	-2.7	426 (350)	425 (343)	-6.4 (-15.9, 3.1)	-1.5
+15	345 (213)	347 (201)	-9.2 (-14.6, -3.9)	-2.6	423 (354)	424 (355)	-8.2 (-18.0, 1.7)	-1.9
+16	348 (216)	351 (205)	-10.0 (-15.5, -4.5)	-2.8	420 (356)	421 (354)	-7.6 (-17.6, 2.4)	-1.8
+17	348 (222)	352 (214)	-10.9 (-16.6, -5.1)	-3.0	414 (375)	416 (380)	-9.5 (-20.1, 1.1)	-2.2

ESM Table 17. Post-hoc analysis. Mean absolute, mean difference with 95% CIs, and relative differences of pension-qualifying incomes, excluding the parental care allowance, in parents of children with type 1 diabetes (exposed) and the population-based matched parental controls (unexposed). Index years 1993-2014. Yearly incomes are reported in €100.

	Mothers				Fathers			
Year (t)	Exposed Mean (SD)	Unexposed Mean (SD)	Mean difference (95% CI)	Relative difference (%)	Exposed Mean (SD)	Unexposed Mean (SD)	Mean difference (95% CI)	Relative difference (%)
-3	221 (137)	215 (146)	-0.7 (-2.3, 0.8)	-0.3	351 (239)	343 (279)	1.1 (-1.7, 3.9)	0.3
-2	230 (145)	223 (153)	-0.4 (-1.6, 0.9)	-0.2	361 (255)	353 (284)	0.5 (-1.7, 2.7)	0.1
-1	241 (154)	233 (160)	0	0	371 (265)	363 (297)	0	0
Index	246 (155)	246 (170)	-7.3 (-8.6, -6.1)	-2.9	381 (281)	375 (308)	-2.3 (-4.7, 0.0)	-0.6
+1	255 (163)	259 (173)	-11.5 (-13.1, -10.0)	-4.3	391 (285)	387 (316)	-3.5 (-6.3, -0.8)	-0.9
+2	266 (169)	272 (180)	-14.0 (-15.8, -12.3)	-5.0	399 (281)	398 (331)	-6.3 (-9.5, -3.1)	-1.6
+3	280 (182)	285 (186)	-13.1 (-15.2, -11.1)	-4.5	410 (296)	408 (338)	-6.2 (-9.7, -2.7)	-1.5
+4	292 (187)	298 (191)	-13.7 (-15.9, -11.6)	-4.5	420 (300)	418 (340)	-5.3 (-8.9, -1.7)	-1.3
+5	304 (197)	310 (201)	-13.0 (-15.3, -10.6)	-4.1	429 (311)	426 (344)	-5.2 (-9.0, -1.3)	-1.2
+6	316 (212)	321 (204)	-12.6 (-15.2, -10.0)	-3.8	436 (324)	434 (352)	-5.1 (-9.3, -0.9)	-1.2
+7	327 (211)	331 (209)	-12.1 (-14.8, -9.5)	-3.6	445 (337)	441 (385)	-3.8 (-8.2, 0.7)	-0.8

ESM Table 18. Post-hoc analysis. Mean absolute, mean difference with 95% CIs, and relative differences of pension-qualifying incomes, excluding the parental care allowance, in parents of children with type 1 diabetes (exposed) and the population-based matched parental controls (unexposed). Index years 1993-2004. Yearly incomes are reported in €100.

	Mothers				Fathers			
Year (t)	Exposed Mean (SD)	Unexposed Mean (SD)	Mean difference (95% CI)	Relative difference (%)	Exposed Mean (SD)	Unexposed Mean (SD)	Mean difference (95% CI)	Relative difference (%)
-3	182 (101)	176 (111)	-1.5 (-3.6, 0.7)	-0.8	301 (178)	293 (221)	1.2 (-3.0, 5.4)	0.4
-2	189 (110)	183 (116)	-0.6 (-2.3, 1.1)	-0.3	312 (220)	304 (231)	1.2 (-1.7, 4.1)	0.4
-1	199 (118)	191 (121)	0	0	323 (235)	315 (260)	0	0
Index	204 (117)	202 (126)	-5.7 (-7.4, -4.1)	-2.7	334 (268)	327 (258)	-0.1 (-3.4, 3.1)	-0.0
+1	209 (124)	214 (133)	-12.0 (-14.2, -9.9)	-5.4	344 (247)	338 (267)	-0.2 (-4.4, 4.0)	-0.1
+2	217 (129)	226 (138)	-15.4 (-17.8, -13.0)	-6.6	350 (233)	350 (268)	-6.0 (-11.6, -0.3)	-1.7
+3	228 (134)	237 (144)	-16.3 (-18.9, -13.6)	-6.7	360 (234)	361 (278)	-7.4 (-13.3, -1.6)	-2.0
+4	238 (138)	248 (151)	-16.8 (-19.6, -13.9)	-6.6	370 (252)	371 (282)	-6.8 (-12.9, -0.7)	-1.8
+5	252 (146)	260 (154)	-14.8 (-17.9, -11.7)	-5.6	379 (242)	380 (295)	-7.6 (-13.4, -1.9)	-2.0
+6	262 (148)	270 (156)	-14.5 (-17.7, -11.3)	-5.2	386 (247)	388 (300)	-8.3 (-14.4, -2.2)	-2.1
+7	272 (151)	280 (168)	-14.8 (-18.1, -11.5)	-5.2	394 (254)	395 (315)	-7.3 (-13.8, -0.9)	-1.8
+8	281 (159)	288 (164)	-14.4 (-18.0, -10.9)	-4.9	399 (268)	401 (317)	-7.7 (-14.4, -1.1)	-1.9
+9	290 (162)	298 (174)	-14.8 (-18.4, -11.2)	-4.9	408 (306)	407 (352)	-5.3 (-13.1, 2.4)	-1.3
+10	300 (166)	308 (174)	-14.8 (-18.5, -11.0)	-4.7	413 (297)	412 (350)	-5.2 (-12.9, 2.5)	-1.2
+11	310 (173)	317 (178)	-13.5 (-17.5, -9.5)	-4.2	418 (304)	417 (336)	-6.0 (-13.9, 1.9)	-1.4
+12	319 (180)	326 (183)	-13.5 (-17.8, -9.3)	-4.1	424 (322)	421 (329)	-4.1 (-12.5, 4.4)	-0.9
+13	329 (192)	334 (192)	-12.2 (-16.9, -7.6)	-3.6	425 (338)	424 (340)	-6.2 (-15.1, 2.7)	-1.4
+14	334 (197)	341 (196)	-13.4 (-18.3, -8.6)	-3.9	425 (350)	425 (343)	-6.9 (-16.4, 2.6)	-1.6

	Mothers				Fathers			
Year (t)	Exposed Mean (SD)	Unexposed Mean (SD)	Mean difference (95% CI)	Relative difference (%)	Exposed Mean (SD)	Unexposed Mean (SD)	Mean difference (95% CI)	Relative difference (%)
+15	341 (213)	346 (201)	-11.6 (-16.9, -6.3)	-3.3	422 (354)	424 (355)	-8.5 (-18.3, 1.4)	-2.0
+16	345 (216)	350 (205)	-11.4 (-16.9, -5.9)	-3.2	419 (356)	421 (354)	-7.7 (-17.7, 2.3)	-1.8
+17	347 (222)	351 (214)	-11.5 (-17.3, -5.8)	-3.2	413 (375)	416 (380)	-9.5 (-20.2, 1.1)	-2.3

ESM Figure 1. Post hoc analysis. Yearly effect estimates from the difference-in-differences analysis (mean differences and 95% CIs) of work-related incomes in a) mothers, and b) fathers of children with type 1 diabetes by the number of children (0-17 years) residing in the parental household the year preceding the index year.

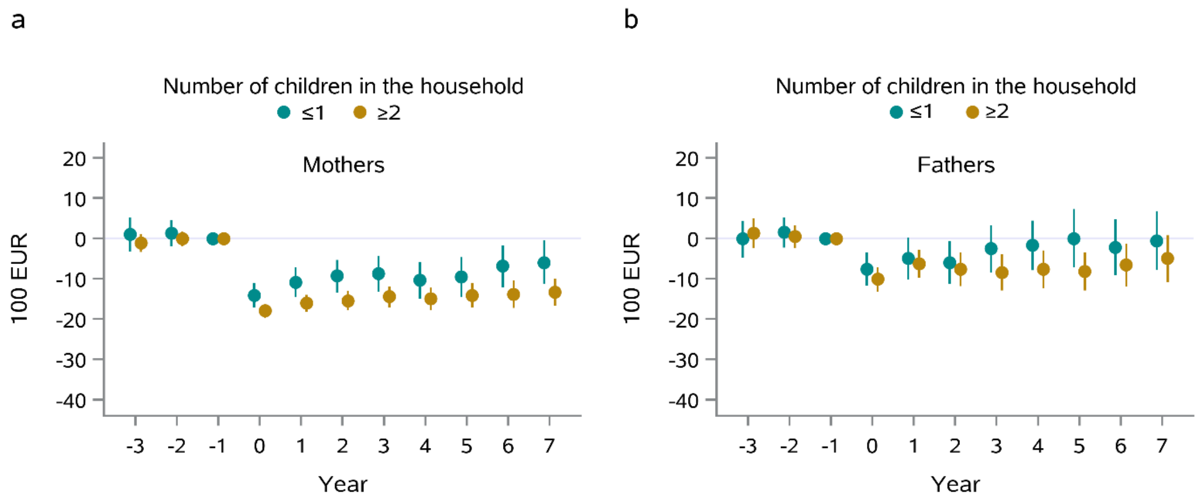

ESM Figure 2. Yearly effect estimates from the difference-in-differences analysis (mean differences and 95% CIs) of pension-qualifying incomes in mothers (a) and fathers (b) of children with type 1 diabetes, with and without parental care allowance. Index years 1993-2014. Incomes are reported in €100.

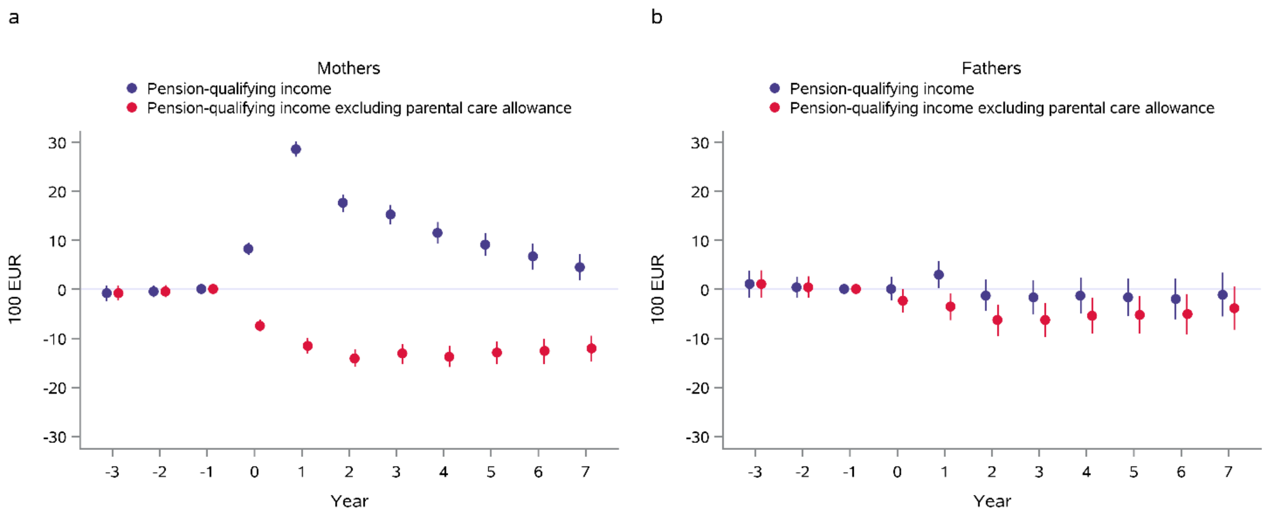

ESM Figure 3. Sensitivity analysis. Top panels: Yearly effect estimates from the difference-in-differences analysis (mean differences and 95% CIs) of work-related incomes in mothers (a) and fathers (b) of children with type 1 diabetes. Bottom panels: Yearly effect estimates from the difference-in-differences analysis (mean differences and 95% CIs) of pension-qualifying incomes in mothers (c) and fathers (d) of children with type 1 diabetes, with and without parental care allowance. Index years 1997-2014. Incomes are reported in €100.

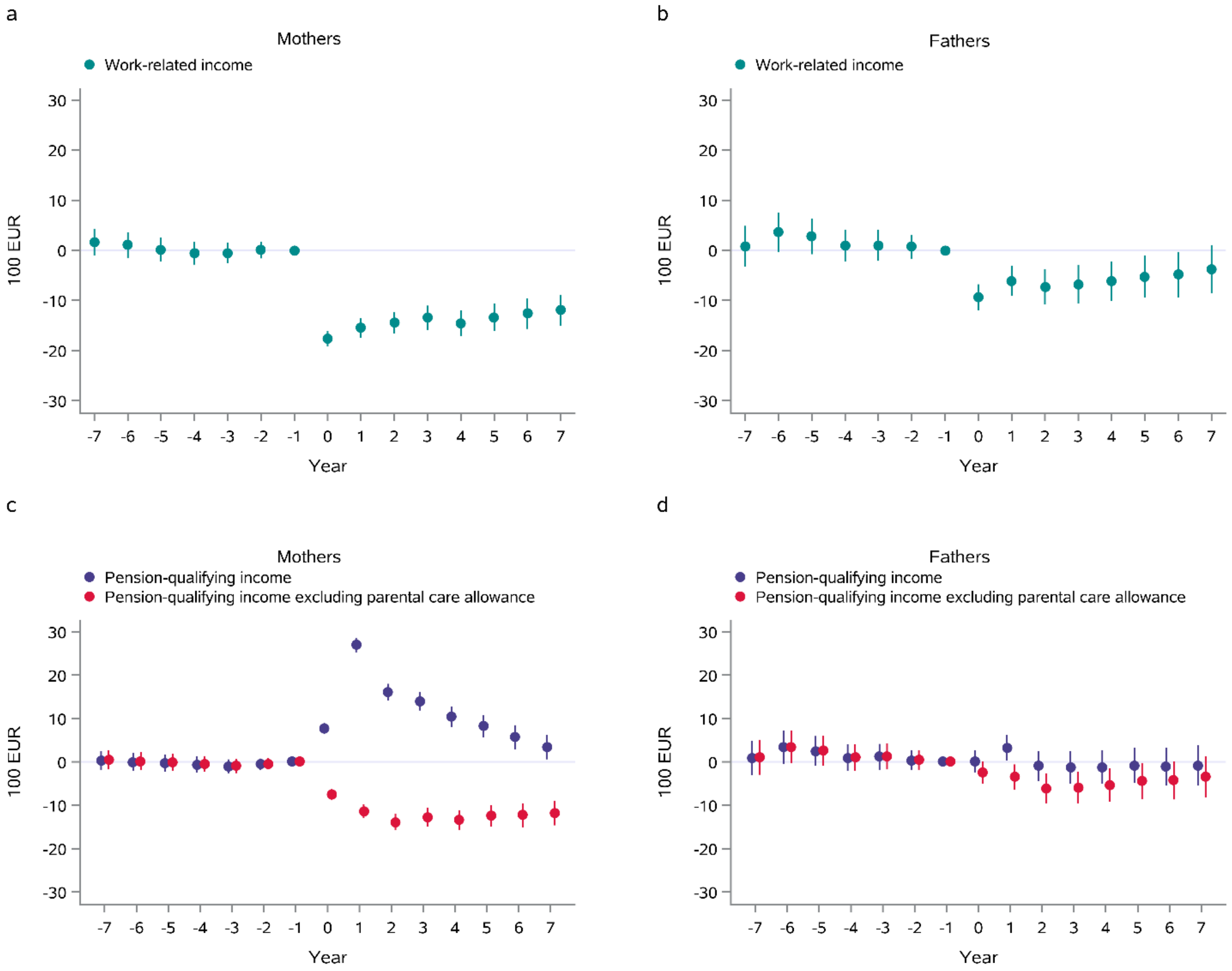

REFERENCES

[1] SWEDIABKIDS Nationella Diabetesregistret - barn och ungdomsdiabetes www.ndr.nu (2020) Årsrapport: 2020 ÅRS RESULTAT.
